# Starvation-induced collective behavior in *C. elegans*

**DOI:** 10.1038/srep10647

**Published:** 2015-05-27

**Authors:** Alexander B. Artyukhin, Joshua J. Yim, Mi Cheong Cheong, Leon Avery

**Affiliations:** 1 Department of Physiology and Biophysics, Virginia Commonwealth University, Richmond, VA 23298, USA; 2 Department for Evolutionary Biology, Max Planck Institute for Developmental Biology, Tubingen, Germany; 3 Current address: Boyce Thompson Institute for Plant Research, Ithaca, NY 14853, USA; 4 Current address: Department of Chemical and Systems Biology, Stanford University, Stanford, CA 94305, USA; 5 Current address: Department of Pharmacology, University of Texas Southwestern Medical Center, Dallas, TX 75390, USA

## Abstract

We describe a new type of collective behavior in *C. elegans* nematodes, aggregation of starved L1 larvae. Shortly after hatching in the absence of food, L1 larvae arrest their development and disperse in search for food. In contrast, after two or more days without food, the worms change their behavior—they start to aggregate. The aggregation requires a small amount of ethanol or acetate in the environment. In the case of ethanol, it has to be metabolized, which requires functional alcohol dehydrogenase *sodh-1*. The resulting acetate is used in de novo fatty acid synthesis, and some of the newly made fatty acids are then derivatized to glycerophosphoethanolamides and released into the surrounding medium. We examined several other *Caenorhabditis* species and found an apparent correlation between propensity of starved L1s to aggregate and density dependence of their survival in starvation. Aggregation locally concentrates worms and may help the larvae to survive long starvation. This work demonstrates how presence of ethanol or acetate, relatively abundant small molecules in the environment, induces collective behavior in *C. elegans* associated with different survival strategies.

Humans are a social species, which naturally inclines us to consider the complex structure of our own society as a crown of evolutionary development. But various forms of sociality have emerged and disappeared countless times throughout evolution. There are many puzzling examples of closely related species in which some are social while others are solitary^1^. We also know organisms that dramatically change their social behavior in response to environmental conditions^2^. Animals live in complex and dynamic environments. Some environmental changes, such as seasonal variations in temperature and food availability, are more predictable, others appear more random. In most cases, the mechanisms that determine how an uncertain and fluctuating environment affects individual behaviors and how new functionalities emerge at a population level as a result of these interactions are far from understood. *C. elegans* does not immediately come to mind when one thinks of social species. Yet, these animals are capable of associative learning and memory, they interact with each other through mating, social foraging, population density sensing, and participate in other neuron-mediated behaviors^3^,^4^. Advantages of a “model” organism, such as availability of mutant and transgene libraries, render the task of deciphering molecular mechanisms of social interactions practically achievable in this animal.

After hatching from an egg, *C. elegans*, similarly to other nematodes, goes through four larval stages, L1 to L4, before becoming an adult^5^. When eggs hatch in the absence of food, newly hatched L1 larvae arrest their development and can survive in this state for weeks. Once food becomes available, they exit the arrest and resume normal development^6^,^7^. Working with *C. elegans*, we have noticed aggregation of starved L1s on old agar plates after the worms have exhausted the food (typically in the lab, *E. coli*). A possible explanation for this phenomenon could be that larvae aggregate around worm corpses in their search for food, being attracted by the smell of decomposing worm bodies. If this were true, one would expect that starved (but living) larvae transferred to a fresh plate should not aggregate. Contrary to this expectation, we found that after brief initial dispersal starved L1 larvae aggregated on fresh agar plates as efficiently as they did on old, exhausted plates. This result rules out any necessity for worm corpses in L1 aggregation and suggests a more interesting mechanism, in which L1s interact with each other. It is the mechanism of this new collective behavior that we seek to explore in this paper.

## Results

### Starved *C. elegans* L1 worms aggregate

**Figure 1:**
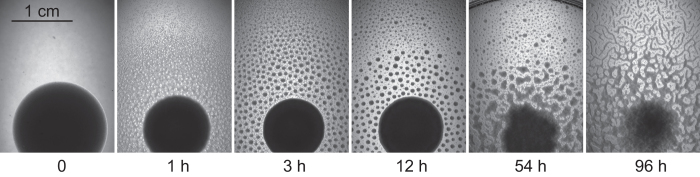
Time course of *C. elegans* L1 aggregation. One million 3 day starved wild-type L1 larvae were thoroughly washed, dispersed in 100 μl of M9 buffer, and pipetted into the center of a clean 6 cm NGMSR plate at time zero (dark circular area in the lower half of the images).

To avoid ambiguity in age, composition, and potential contamination of exhausted plates, we performed all aggregation experiments on clean, foodless plates onto which we pipetted a defined number of L1 larvae prestarved in a buffer for a specific amount of time. We thoroughly washed the worms before pipetting on the plate to remove any soluble substances that they may have secreted up to this point. (We know there are many^8^.) We observed three distinct stages of worm behavior after L1 larvae were pipetted on a clean plate (Fig. 1). During the first hour worms disperse from the starting region and gradually fill the entire plate (Fig. 1, 1 h). There is no sign of any order or correlation among worms during this time, local density fluctuations quickly appear and disappear. After an hour or two small fluctuations in worm density suddenly amplify and give rise to an array of circular aggregates (Fig. 1, 3 h), which is then metastable for at least 24 h (Fig. 1, 12 h). By “metastable”, we mean that once formed the aggregates do not dissociate, but neighboring aggregates may occasionally merge (Fig. S1). After 1-4 days circular aggregates start to lose their shape (Fig. 1, 54 h) and aggregate morphology transitions to stripes (Fig. 1, 96 h). The timing of this change in aggregate morphology, which we hereafter refer to as disks-to-stripes transition, depends on worm density on the plate—the more worms, the faster it happens. In this paper we mostly focus on the second stage of L1 aggregation—formation of circular aggregates. As expected for many-body interactions, L1 aggregation depends critically on worm density. A threshold density is required for aggregation, which suggests that L1 aggregation is a fairly weak interaction (e.g. compared to sexual attraction^9^). There is no visible interaction between two L1s crawling on a plate, even when they run into each other. Yet, at large numbers random fluctuations can bring enough worms together and nucleate an aggregate. We observed aggregation only after L1 larvae had been prestarved for at least 2 days. L1s within 24 h of hatching disperse on the plate and do not aggregate. We know that L1s adjust their transcriptional program quickly after hatching without food^10^. Apparently, just the fact that the worms are starved and developmentally arrested is not sufficient to make them aggregate. It requires longer starvation.

### L1 aggregation is different from known examples of *C. elegans* aggregation

Several examples of *C. elegans* aggregation have been described in the literature. One of them is mediated by indole-containing ascarosides^11^,^12^. We examined the behavior of starved *daf-22* L1s, which lack all ascarosides commonly detected in wild type^12^. *daf-22* encodes the peroxisomal 3-keto-acyl-CoA thiolase, which is necessary for peroxisomal β-oxidation of fatty acids and ascaroside biosynthesis^13^,^14^,^15^. L1s of *daf-22* mutants aggregated similarly to wild type (Fig. S2). Another known aggregation behavior is modulated by NPR-1 and ambient oxygen concentration^16^,^17^,^18^. Aggregation results from the worm’s preference for lower than ambient oxygen levels and the effect disappears at [O_2_] < 10%. We tested L1 aggregation of wild-type worms in hypoxic conditions and found little effect of [O_2_] on aggregation down to 1% (Fig. S3). From these results we infer that the mechanism of starved L1 aggregation is different from known examples of social behavior in *C. elegans*.

### Ethanol is required for L1 aggregation

**Figure 2:**
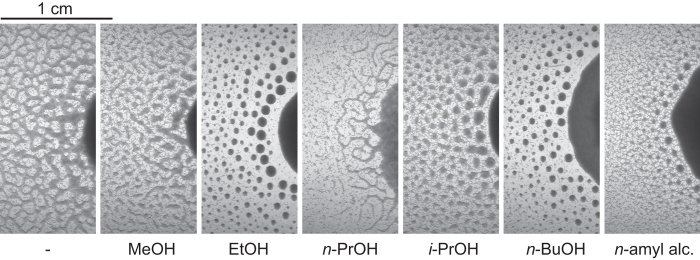
Even-chain alcohol is required for L1 aggregation. Images obtained 23 h after pipetting 3 day starved L1s to agar plates supplemented with 17 mM of various alcohols (10^6^ L1 larvae per plate). The leftmost image is a control without alcohol.

For convenience reasons we performed initial aggregation experiments on unseeded Nematode Growth Medium Streptomycin Resistant (NGMSR) plates. At the time we assumed that the substrate surface plays only a passive role in the aggregation process and its composition is not important. When we tried to repeat these studies on pure agar plates, we were surprised to discover that worms failed to aggregate. Instead, they formed a stripe pattern resembling the one observed after several days on NGMSR plates (Fig. 1, 96 h), but they never formed circular aggregates. Intrigued by this result, we eliminated NGMSR components one by one and found that 0.1% (v/v) ethanol is necessary and sufficient for the aggregation. Ethanol comes from the stock solution of cholesterol that goes into NGMSR. Aggregation requires none of the other NGMSR components and agar can be easily substituted by agarose. Ethanol concentration study showed that as little as 0.01% ethanol is enough to elicit stable L1 aggregation (Fig. S4). We note that this ethanol concentration is far lower than typically required for reduced motility and other behavioral phenotypes associated with ethanol intoxication^19^,^20^. To determine if other short-chain alcohols would also support the aggregation, we tested several alcohols and found that only even-chain molecules elicited this response (ethanol and *n*-butanol), whereas odd-chain alcohols did not (methanol and *n*-propanol) (Fig. 2). This result suggests that it is likely not a physical property of the alcohol molecule that is important for the aggregation but perhaps something related to its metabolism.

### Disks-to-stripes transition is reversible

**Figure 3:**
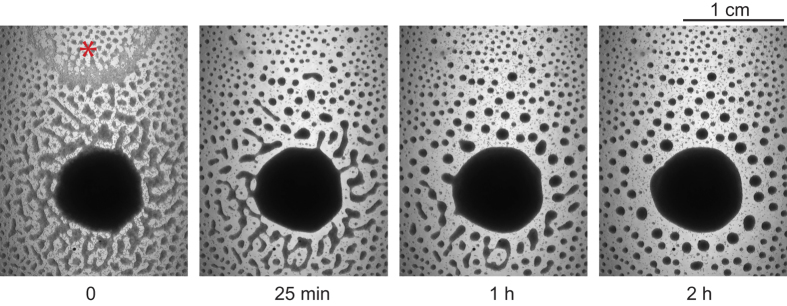
Addition of ethanol quickly induces L1 aggregation. 5 μl of ethanol was added to one side of a 6 cm plate containing L1s (red asterisk).

We then hypothesized that the density dependence of the disks-to-stripes transition mentioned earlier may result from the fact that more worms on the plate consume a given amount of alcohol faster. We reasoned that once all alcohol is exhausted, there is no driver for aggregation and circular aggregates transition to stripes. (See Discussion for what may determine the aggregate morphology.) To test this idea, we added a small amount of ethanol to one side of each of two L1 plates. The first plate originally had 0.1% ethanol during aggregation but was incubated for enough time to allow for the disks-to-stripes transition to occur. The second plate did not originally have ethanol and the only aggregates that formed there had the stripe shape from the beginning. In both cases addition of ethanol caused rapid rounding and contraction of stripe aggregates, and eventually all aggregates became circular (Fig. 3 and data not shown). The same process, though at a slower rate, happened on the L1 plate that was aged for more than a week after the initial aggregation experiment (Fig. S5). This result proves that the worms in the stripes are alive and suggests that the disks-to-stripes transition is not due to the extended starvation or general sickening of the worms. Rather, it is caused by depletion of ethanol in the substrate and can be quickly reversed by addition of small amount of ethanol to the plate. This implies that ethanol is not enough to just initiate the aggregation, it must be continuously supplied to maintain the aggregated state. Quick reversal of this transition by adding ethanol also argues that worm metabolites accumulated in the agar during the aggregation do not interfere with the aggregation process.

### Ethanol metabolism is important for L1 aggregation

**Figure 4:**
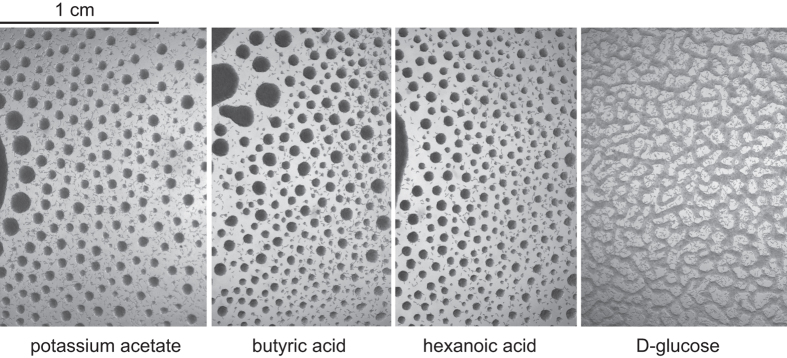
Short-chain fatty acids support L1 aggregation. L1s aggregate in the presence of 17 mM potassium acetate, butyric, or hexanoic acids, but not glucose.

How does alcohol lead to L1 aggregation? Is it alcohol itself or its metabolites that are important? To address these questions, we substituted ethanol with several carboxylic acids and found that presence of either acetic, butyric, or hexanoic acids supports L1 aggregation similarly to ethanol (Fig. 4). Addition of these acids can also reverse the disks-to-stripes transition. Interestingly, propionic acid had no effect, which is consistent with the difference between even- and odd-chain alcohols mentioned earlier. The effect of acids reinforces the hypothesis that it is not alcohol but its downstream metabolites that are necessary for the aggregation. Alternatively, it could be energy gained during ethanol and acetate oxidation that drives aggregation of starved worms. If this were so, one would expect any molecule that has nutritional value for worms to elicit aggregation behavior. However, this is not the case. Supplementation of agar with either peptone or glucose (but without ethanol) is insufficient for the aggregation to occur (Fig. 4). At the same time, we know that both amino acids and D-glucose can extend L1 starvation survival^8^, which implies that worms can extract these molecules from the medium and metabolize them. Thus, the aggregation of starved L1s is not a response to the presence of any nutrient. It is a specific response to alcohol metabolites, most probably carboxylic acids.

### *sodh-1* is required for L1 aggregation

**Figure 5:**
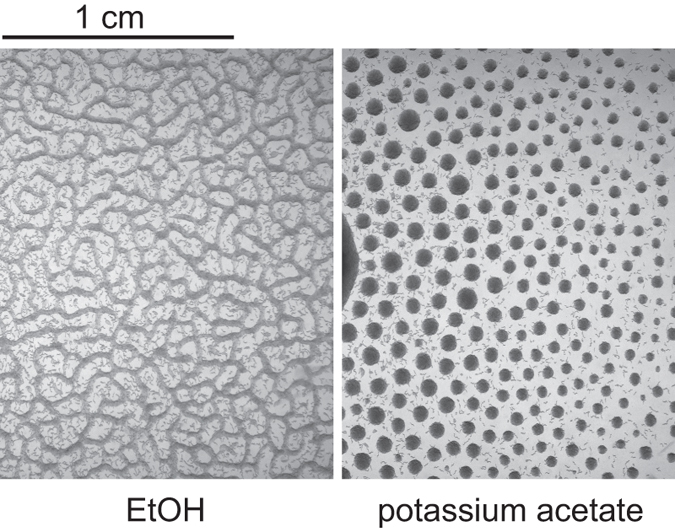
*sodh-1* is required for aggregation in the presence of ethanol. *sodh-1(ok2799)* L1s fail to aggregate on ethanol-containing plates but aggregate normally in the presence of 17 mM potassium acetate. The other *sodh-1* alleles, *bet20* and *tm2829*, showed the same result.

If our previous conclusion is true, one would predict that a mutant incapable of metabolizing ethanol should not aggregate. The canonical pathway of alcohol metabolism consists of two steps—the first is oxidation of the alcohol to an aldehyde by alcohol dehydrogenase, which is followed by oxidation of aldehyde to acid catalyzed by aldehyde dehydrogenase. *C. elegans* has several aldehyde dehydrogenase genes (*alh*)^20^,^21^. For many of them specific functions are unknown and RNAi knockdown of individual genes had no visible phenotype^20^, probably due to redundancy. The situation is clearer with the alcohol dehydrogenase. It has been demonstrated that the alcohol dehydrogenase enzyme encoded by the *sodh-1* gene is required for the first step of ethanol metabolism in *C. elegans*^20^,^22^. We tested three *sodh-1* alleles and found that worms carrying any of them did not aggregate in the presence of 0.1% ethanol (Fig. 5 and data not shown). At the same time, all three alleles showed normal aggregation in the presence of acetate (Fig. 5). These results confirm our hypothesis that alcohol metabolism is important for the aggregation. When we bypass the block in the metabolic pathway by supplementing a downstream metabolite, the aggregation proceeds normally. This again highlights the role of carboxylic acids or their downstream metabolites, rather than alcohols themselves, in L1 aggregation.

### New ethanol/acetate derived metabolites

**Figure 6:**
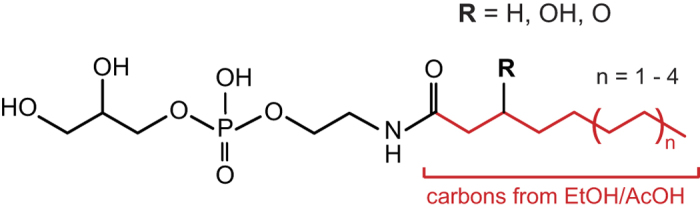
Glycerophosphoethanolamides are produced from ethanol. A general formula of ethanol/acetate specific metabolites detected in L1 medium. Ethanol-derived carbons are shown in red.

Based on the observed difference between even- and odd-chain molecules, we assumed that longer acids, such as butyric and hexanoic, are first broken down to acetate. Therefore we focused on acetate and asked what it does in the animal. Acetate, or its activated form, acetyl-CoA, can participate in numerous processes in the cell, such as fatty acid synthesis, Krebs cycle, and protein acetylation to name a few. To gain insight into acetate fate in starved L1 worms, we performed differential untargeted LC-MS analysis^23^,^24^ of metabolome extracts from worms supplemented with ethanol or acetate versus an untreated control. While ethanol and acetate samples had similar LC-MS profiles, we discovered tens of new peaks in those samples that were absent in untreated controls. MS/MS analysis of these peaks and comparison with synthetic standards allowed us to assign structures for many of the new metabolites. More than half of the ethanol/acetate-specific metabolites that we could detect in L1 samples belong to a class of glycerophosphoethanolamides^25^,^26^ and have even-number acyl chains with 8 to 14 carbons (Fig. 6, Fig. S6-S25). Distributions of acyl chain lengths of glycerophosphoethanolamides were similar between ethanol and butanol (or butyrate) fed worms. This is consistent with our assumption that longer acids are first broken down to acetate, which is then used in de novo fatty acid synthesis. Analysis of the metabolome of L1s starved in the presence of ^13^C_2_-labeled ethanol revealed that 95% of carbon atoms in the fatty acid chains of these glycerophosphoethanolamides are derived from ethanol (Fig. 6, Fig. S6-S8, S11-S13). While it has been known that starved L1s incorporate ethanol, probably in the form of Ac-CoA, in their fatty acids^21^,^27^, we were surprised to find large amounts of fatty acid derivatives that were built almost exclusively from ethanol carbons. Furthermore, comparing extracts of worm conditioned medium and worm pellets we found that worms preferentially released these newly made fatty acid derivatives into the medium.

### Elusive aggregation cue

**Figure 7:**
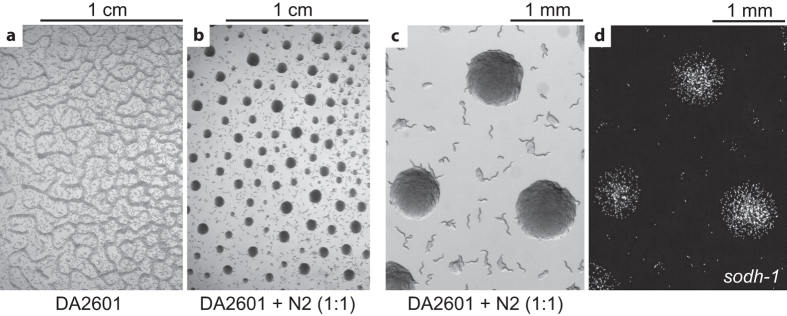
*sodh-1* L1s participate in wild-type aggregates. *sodh-1(tm2829)* L1s expressing tdtomato fluorescent protein do not aggregate (**a**) but a 1:1 mixture with wild-type (N2) worms does (**b,c**). (**d**) A fluorescence image of the same area as shown in (**c**) illustrates that *sodh-1* larvae are mostly in the aggregates. We obtained similar results with other alleles of *sodh-1* and with different ratios of *sodh-1*:N2, varying from 1:4 to 3:1.

We then asked if glycerophosphoethanolamides or some other ethanol/acetate-specific metabolites could be the chemical signals that mediate L1 aggregation. Considering that ethanolamides are generally bioactive molecules in various species, including *C. elegans*^28^,^29^,^30^,^31^, we thought it was plausible. Nevertheless, all our attempts to detect any activity of the conditioned medium in bioassays focused on the aggregation phenotype failed. (See Methods for brief description of the assays.) We cannot exclude the possibility that the signal is short-lived and it does not take much imagination to come up with other possible explanations for this failure, but so far we have no positive evidence demonstrating that the aggregation involves a diffusible cue. To approach this question from another perspective, we examined aggregation in mixtures of wild type and *sodh-1* worms in the presence of ethanol. Since *sodh-1* L1s cannot metabolize ethanol, they do not aggregate by themselves under these conditions. We reasoned that if wild-type worms release an aggregation signal, the mutant worms in the mixture may be able to respond to it and participate in the aggregates. To distinguish between the two genotypes, we introduced a fluorescent marker into *sodh-1* background. We found that a 1:1 mixture of wild type and *sodh-1* L1s forms aggregates indistinguishably from wild type alone and *sodh-1* mutant worms are found mostly in the aggregates (Fig. 7). This result not only shows that the presence of wild-type animals enables naive *sodh-1* worms to aggregate and hints at the presence of a worm-released signal but also suggests that the effect of ethanol metabolism on the internal metabolic state of the animal is not important for the aggregation. A potential caveat in this experiment is that it is possible that acetate produced from ethanol metabolism in wild-type worms leaks out and that is what is causing *sodh-1* L1s to aggregate.

### L1 aggregation in other *Caenorhabditis* species

**Figure 8:**
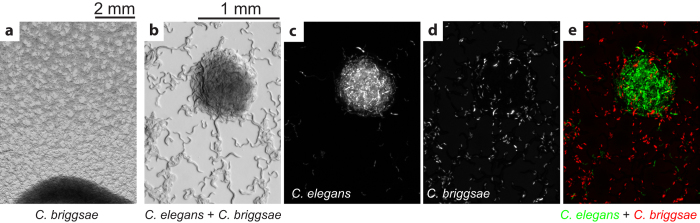
*C. briggsae* L1s do not aggregate. (**a**) Starved *C. briggsae* AF16 L1 worms show no sign of aggregation 15.5 h after being put on a NGMSR plate. (**b**) A bright field image of a mixture of *C. elegans* L1s expressing GFP (PD4792) and *C. briggsae* L1s expressing dsRed (JU1018). Fluorescence images (**c**, **d**) allow the two species to be distinguished and demonstrate that *C. briggsae* worms do not participate in the aggregates. (**e**) Overlay of two fluorescence channels. Images b-e correspond to the same area.

How general is the L1 aggregation phenomenon? We examined behavior of *C. briggsae*, another species from the genus *Caenorhabditis*^32^, and found that these worms do not aggregate under the same conditions that *C. elegans* L1s do (Fig. 8a). When we mixed *C. elegans* and *C. briggsae* worms expressing different fluorescent markers, *C. elegans* aggregated as if they were by themselves but *C. briggsae* did not participate in these aggregates (Fig. 8b-e). This result is strikingly different from the N2 + *sodh-*1 aggregation described above (Fig. 7) and demonstrates that *C. briggsae* are unable to respond to the aggregation cue produced by worms from a different species. *C. elegans* and *C. briggsae* have many differences^33^ and one of them is the density dependence in L1 starvation survival. We previously reported that *C. elegans* L1s survive starvation longer when they are at high worm density, whereas for *C. briggsae* worm density has no effect on survival^8^. We hypothesize that L1 aggregation and the density dependence are related phenomena—the aggregation could be a way to locally increase worm density, which would lead to longer survival. To test this idea, we examined L1 behavior in two other *Caenorhabditis* species, *C. remanei* and *C. sp. 11*, and again found a correlation between their aggregation and density dependence. L1 larvae of species that aggregate have strong density dependence in survival, while the ones that do not aggregate show no density effect. Although aggregation and density dependence phenomena may be physiologically related, their molecular mechanisms are at least partly different. The density dependence does not require presence of ethanol or acetate and *sodh-1* L1s have wild-type density dependence.

## Discussion

Many people working with *C. elegans* have probably observed aggregation of L1 larvae on starved plates. The nature and mechanism of this phenomenon appears to be far less trivial than we initially thought. First, it requires long (2+ days) starvation. Arrested L1s within the first day after hatching disperse and do not aggregate. Transcriptional and metabolic studies show clear differences between fed and starved L1s within hours after hatching, if not faster, so when worms hatch in the absence of food they know it right away^10^,^34^. Apparently, there is a change in behavior that happens later. We speculate that worm behavior in early starvation is mostly governed by the search for food. This leads to dispersal and exploration behavior. As starvation progresses and the chance diminishes that food will be found quickly, it does not make sense to spend time and energy searching and other strategies should be employed. One would be better off by minimizing unnecessary expenditures and preparing for a long battle for survival—worms aggregate.

Second, L1 aggregation requires a small amount of ethanol in the plates. More precisely, it requires ethanol to be metabolized and it is acetate, acetyl-CoA, or molecules downstream that are likely involved. Although it is purely due to the fact that early *C. elegans* researchers used ethanol to prepare cholesterol stock^35^ that we discovered L1 aggregation in the lab, we believe that this effect may have ecological importance. *C. elegans* is a free-living nematode. In the wild, it forages for blooms of bacteria that arise capriciously in rotting fruit and other decomposing material^36^. It is reasonable to assume that worms encounter 0.1% or higher concentrations of ethanol in their natural environment^37^.

How do worms interact with each other to form aggregates? These interactions could be of chemical or mechanical nature, or both, but a requirement for specific molecules (ethanol or acetate) speaks in favor of the chemical nature of the aggregation signal. The process can be illustrated with an active walker model^38^. This model relies on chemotaxis towards an attractant that worms themselves secrete, which creates a positive feedback loop. In this paradigm, small random fluctuations in initially uniform worm density amplify and eventually lead to an array of aggregates. The model would work similarly in the case of local decomposition of a uniformly distributed repellant. This is the scenario for worm aggregation induced by avoidance of high oxygen levels^18^. We also cannot exclude more complex mechanisms, such as mechanosensation dependent on a chemical signal. We screened several sensory mutants (*che-2*, *osm-6*, *ttx-1*, *mec-3*, *mec-4*, *asic-1; mec-10*) but so far could not find one that was reproducibly deficient in L1 aggregation.

Shape instabilities and transitions between circular droplets and stripes have been observed and studied in physical sciences for years^39^. Typical examples are Langmuir monolayers at air-water interface and ferrofluids in magnetic fields^40^. In these systems domain shape is determined by an interplay between long-range attraction (e.g. surface tension) and short-range repulsion (e.g. electrostatic interactions)^39^,^41^,^42^. As the balance between these interactions shifts, a domain will undergo a sequence of shape transitions. More circular shapes correspond to stronger attractive forces, while weaker attraction results in domain elongation, branching, and formation of labyrinth structures. We speculate that something similar may happen during disks-to-stripes transition in L1 aggregation. In the presence of ethanol or acetate worms are strongly attracted to each other and as a result form tight, circular aggregates. As ethanol or acetate are consumed, the attraction weakens, aggregates start to lose their round shape and gradually elongate into stripes. Even though attraction in the stripes is weaker than in circular aggregates, there still must be some, otherwise worms would disperse as individuals. This residual attraction is independent of ethanol or acetate and we think it may be of a more general nature (e.g. surface tension^43^).

Finally, we found that while starved L1 larvae of some *Caenorhabditis* species aggregate (e.g. *C. elegans*), others do not (e.g. *C. briggsae*). In unfavorable conditions, such as starvation, *C. elegans* appear to become more social than *C. briggsae*^33^. In variable and unpredictable environments genotypic fitness can be maximized either by reducing individual-level variance in fitness or by reducing between*-*individual correlations in fitness (or some combination of the two)^44^. We speculate that aggregating or social species may choose to minimize individual-level variance by having a mechanism that helps them to adjust to unfavorable conditions, in part through collective behavior. Aggregation may also serve other purposes ranging from decreasing surface to volume ratio and using diffusible “public goods” to sharing information about quality of the environment. Nonaggregating species may use dispersal as a strategy to minimize between-individual correlations. Based on this hypothesis, we would predict that lack of aggregation and density dependence in *C. briggsae* and other nonaggregating species implies that their starved L1s hardly ever find themselves at high density in nature and the optimal strategy for them is to disperse and actively look for food. Despite recent progress in comparative ecology of *Caenorhabditis* species^32^,^45^,^46^,^47^, we still do not know enough to understand their apparent divergence in survival strategies.

## Methods

### Worm strains

Worms were cultured routinely on NGMSR plates seeded with *E. coli* strain HB101 and maintained at 20°C^48^. For NGMSR composition see “L1 aggregation assay” below. The wild-type strain was *C. elegans* variant Bristol, strain N2. The following mutant strains were used in this study: DA2579 *sodh-1(ok2799) V*, DA2580 *sodh-1(bet20) V,* DA2581 *sodh-1(tm2829) V,* DA2598 *sodh-1(bet20) V; ntIs8[pm-6::tdtomato],* DA2599 *sodh-1(ok2799) V; ntIs8[pm-6::tdtomato]*, DA2601 *sodh-1(tm2829) V; ntIs8[pm-6::tdtomato]*, PD4792 *mIs11[myo-2::GFP pes-10::GFP gut::GFP] IV*, DR476 *daf-22(m130) II*, RB859 *daf-22(ok693) II*. For other *Caenorhabditis* species we used the following wild type and mutant strains: *C. briggsae* AF16, *C. remanei* EM464, *C. sp. 11* JU1373, *C. briggsae* JU1018 *mfIs42[Ce-sid-2; Ce-myo-2::DsRed]*.

### Worm liquid culture

To obtain enough L1s for aggregation experiments, we grew worms in liquid culture. We started by seeding synchronized L1 larvae obtained by bleaching of 2-3 maintenance plates onto six 10 cm plates at 1500-1800 L1s/plate. 2.5 days later (at 20 °C) we washed worms and eggs off the plates and bleached them (8 ml H_2_O + 2 ml bleach + 0.3 ml 10M NaOH for 6 min). After two washes with M9 buffer eggs were resuspended in 3 ml M9 and allowed to hatch and synchronize (24-34h at 20°C). Liquid cultures were started with synchronized L1 larvae obtained as described above and grown at 22 °C, 220 rpm in S-complete medium supplemented with 2% (w/w) *E. coli* HB101 or K12. For a small scale liquid culture, we inoculated 25 ml S-complete in a 250 ml flask with 7∙10^4^ synchronized L1s and added 1 ml 50% *E. coli* stock suspension. We monitored the worm culture during the next 2 days and added *E. coli* as it became depleted. Bleaching after 60 h growth yielded ca. 10^6^ eggs (100 μl egg pellet). For a large scale liquid culture, we inoculated 250 ml S-complete in a 2 l flask with 7∙10^5^ synchronized L1s obtained from a small scale liquid culture and added 10 ml 50% *E. coli* stock suspension. We monitored the worm culture during the next 2.5 days and added *E. coli* as it became depleted. Bleaching after 60–72 h growth yielded ca. 10^7^ eggs (1 ml egg pellet).

### L1 aggregation assay

Eggs obtained from a liquid culture were allowed to hatch in 10-20 ml of M9 buffer and further starved in M9 for a specific amount of time, typically 48-60 h from the egg preparation (22 °C, 220 rpm shaking). At that time we collected hatched L1s by centrifugation, washed 6 times with M9, and resuspended in the minimal amount of residual M9 to obtain a concentrated L1 suspension. We pipetted 50-100 μl of this suspension (corresponding to 0.5-1 million L1s) onto an unseeded 6 cm agar or agarose plate and video recorded worm behavior at 1 frame/min for the first 12 h and then occasionally for several days (Point Grey Grasshopper GRAS-14S5M camera with Computar MLM3X-MP lens). The assay was performed at room temperature, typically 22-24 °C. Control experiments at 20 and 15 °C confirmed that L1 aggregation is not restricted to higher temperatures. Initial experiments were done on NGMSR plates (per 1 l: 3 g NaCl, 20 g agar, 2.5 g peptone, 1 ml of 5 mg/ml cholesterol in ethanol, 25 ml of 1 M potassium phosphate pH 6, 1 ml of 1 M CaCl_2_, 1 ml of 1 M MgSO_4_, 1 ml of 200 mg/ml streptomycin sulfate, 1 ml of 10 mg/ml nystatin in DMF; the last 5 components were added after autoclaving). Later experiments were performed on agarose plates (per 1 l: 3 g NaCl, 20 g agarose, 25 ml of 1 M potassium phosphate pH 6, 1 ml of 1 M CaCl_2_, 1 ml of 1 M MgSO_4_), to which 17 mM ethanol (0.1% v/v) was added a few hours before the beginning of the aggregation assay (although in our experience autoclaving does not eliminate ethanol from the medium). In the case of acetate, it can be added either before or after pouring plates. The inorganic salts are not really necessary for the aggregation but we added them to buffer the medium and avoid osmotic stress. Fluorescence images were recorded on a Zeiss Discovery V.8 stereo microscope equipped with AxioCam MRm camera.

### Extraction of L1 metabolomes

L1 larvae starved for 48-60 h were prepared as described above. After thorough washing a few million L1s were resuspended in 10 ml of fresh M9. At that time we added ethanol, acetate, or another component of interest (typically, 17 mM), and worms were starved for additional 24 h. After spinning down L1s, the medium and the worm pellet were collected separately, frozen, and lyophilized. We extracted lyophilized L1 conditioned medium (CM) with 5 ml methanol overnight (stirring), removed the solvent on a rotary evaporator, and redissolved the residue in 100 μl methanol for HPLC-MS analysis. To check if ethanol-derived metabolites are produced by starved worms both in liquid and on plates, we extracted agarose from L1 aggregation assay plates 24 h after L1s were transferred there. To do this, we washed L1s from the plate with cold M9 and centrifuged the worms. Agarose from the plate was cut in 1 × 1 cm chunks, combined with the M9 wash, frozen, lyophilized, and extracted as described above. To extract L1 worm pellets, we lyophilized them and homogenized in methanol with a glass tissue grinder.

### HPLC-MS analysis of CM

HPLC-MS and HPLC-MS/MS was performed using an Agilent 1100 Series HPLC system equipped with an Agilent Eclipse XDB-C18 column (4.6 mm x 250 mm, 5 μm particle diameter) connected to a Quattro Ultima mass spectrometer (Micromass/Waters) using a 1:1 split. For HPLC, a 0.1% aqueous acetic acid-acetonitrile solvent gradient was used at a flow rate of 1 ml/min, starting with an acetonitrile content of 5% for 5 min, which was increased to 100% over a period of 40 min. The gradient was followed by propanol-acetonitrile (3:1) wash and reconditioning of the column. We noticed that retention times and especially peak shapes for glycerophosphoethanolamides are very sensitive to cleanliness of the column. Even with the propanol wash after every run performance of the column gradually declined and it was necessary to periodically back-flash it with chloroform (every 10 to 40 runs, depending on the samples). This procedure returned the original performance and dramatically improved the peak shapes. Samples were analyzed by HPLC-ESI-MS in both positive and negative ionization modes using a capillary voltage of 3.5 kV and a cone voltage of 60 V. HPLC-MS/MS screening was performed using argon as collision gas at 2.1 mtorr and 30 eV. CM was either analyzed directly without further processing or extracted with methanol as outlined above.

### CM bioassays

We tested CM in various assays related to L1 aggregation behavior. First, we checked if CM or methanol extract of CM are attractive to starved L1s in a spot attraction assay. We used M9 or methanol extract of M9, respectively, as a control spot and pipetted L1s in the middle. Second, we added CM methanol extract to agarose plates and examined L1 behavior on these plates. In one experiment, we also added ethanol, reasoning that large excess of uniformly distributed attractant from CM should disrupt the aggregation process normally observed in the presence of ethanol. In another experiment, we did not add ethanol to the plates hypothesizing that an active component from CM may substitute for it and thus support the aggregation. We also repeated these experiments with methanol extracts of L1 worm pellets, suspecting that the active component may be attached to worm cuticles and not released in the medium. Third, we tested for the presence of volatile signals. We arranged two agar surfaces so that there is a thin air gap between them and looked for correlation in worm positions on the two surfaces. Finally, trying to demonstrate the presence of a diffusible cue, we partitioned agarose in the plate with filter paper or a nitrocellulose membrane and again looked for correlations in aggregate positions on the two sides of the membrane.

### Organic synthesis

Glycerophosphoethanolamide (**ea#10**, Fig. S7) was synthetized according to a published protocol^26^. Briefly, decanoyl chloride was reacted with an excess of 1,2-dioleoyl-*sn*-glycero-3-phosphoethanolamine with a catalytic amount of triethylamine. N-acylated lipids were hydrolyzed with LiOH and acidified with acetic acid.

## Additional Information

**How to cite this article**: Artyukhin, A. B.* et al.* Starvation-induced collective behavior in *C. elegans.* *Sci. Rep.* **5**, 10647; doi: 10.1038/srep10647 (2015).

## Supplementary Material

Supplementary Information
