# Supplementary material for: Starvation-induced collective behavior in C. elegans
Source: Sci Rep. 2015 May 27;5:10647. doi: 10.1038/srep10647 (PMC4445038; doi:10.1038/srep10647)
Supplement: Supplementary Information [file srep10647-s1.pdf]

Supporting Information for

Starvation-induced collective behavior in *C. elegans*

Alexander B. Artyukhin, Joshua J. Yim, Mi Cheong Cheong, and Leon Avery

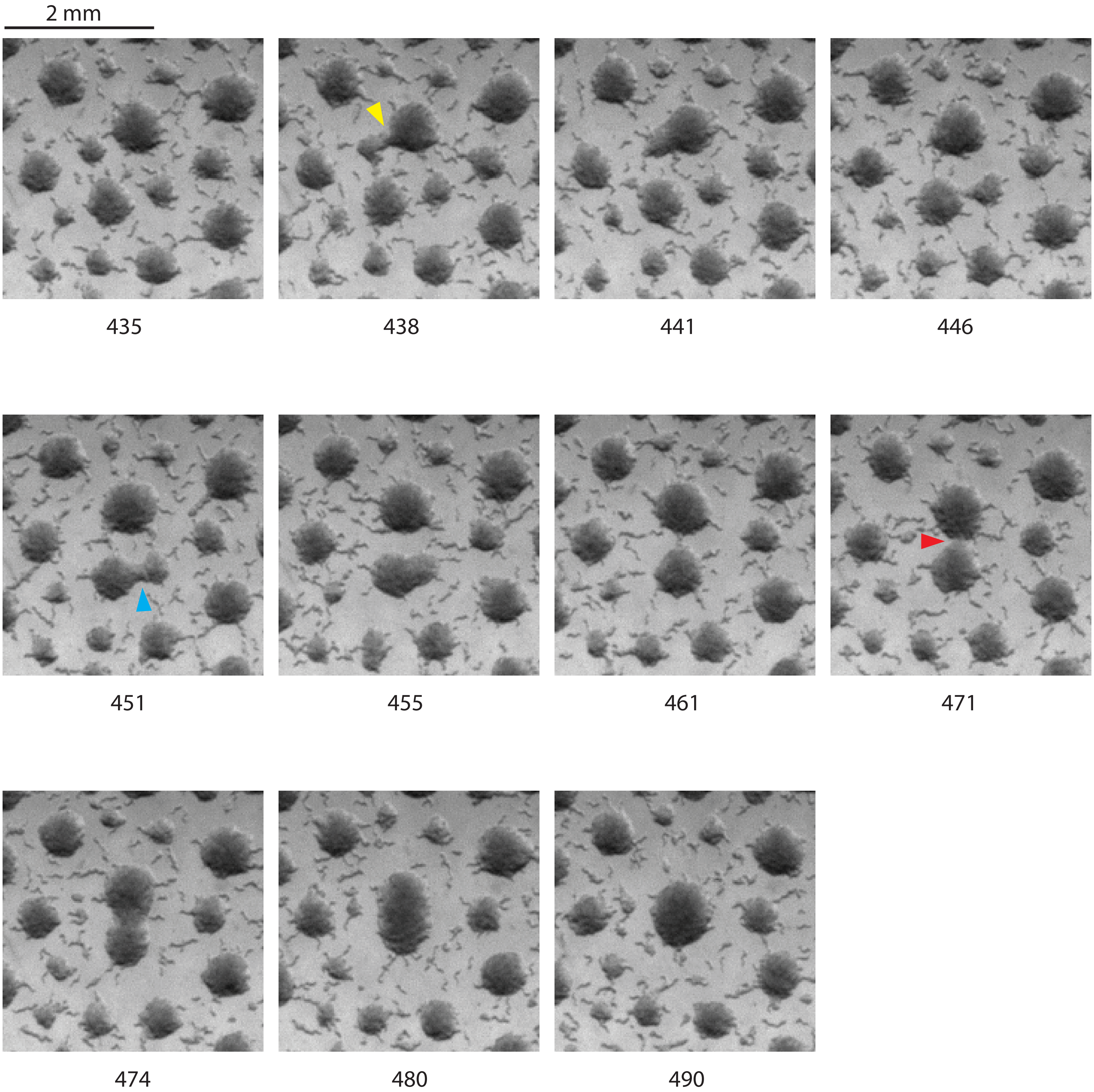

Fig. S1. Time lapse images of L1 aggregation showing merge of neighboring aggregates. Merging aggregates are indicated with colored arrowheads. Numbers under the frames correspond to time points in minutes from the start of the aggregation experiment, i.e. the moment when L1s were pipetted on the plate. All images correspond to the same area.

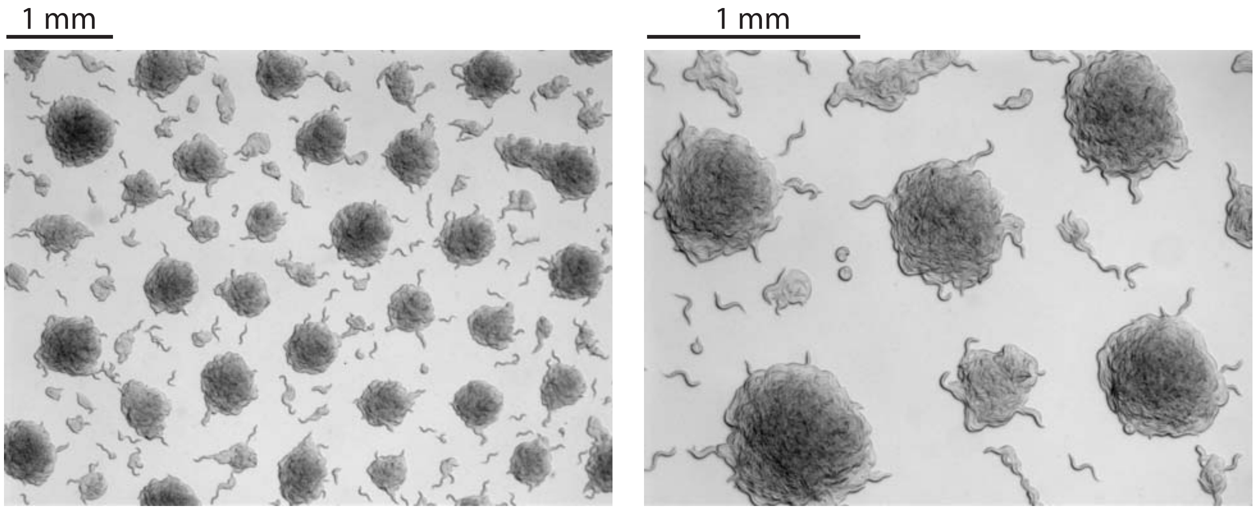

Fig. S2. Aggregation of *daf-22(m130)* L1s demonstrates that ascarosides are dispensible for this process.

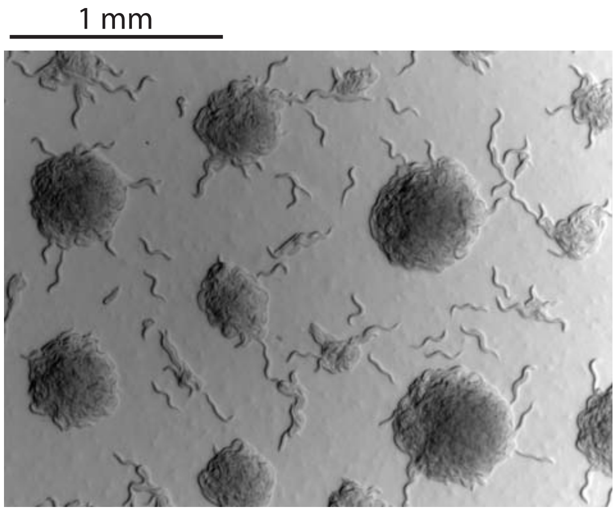

Fig. S3. Aggregation of N2 L1s in 1% oxygen atmosphere. Image was taken 8 h after beginning of the aggregation experiment.

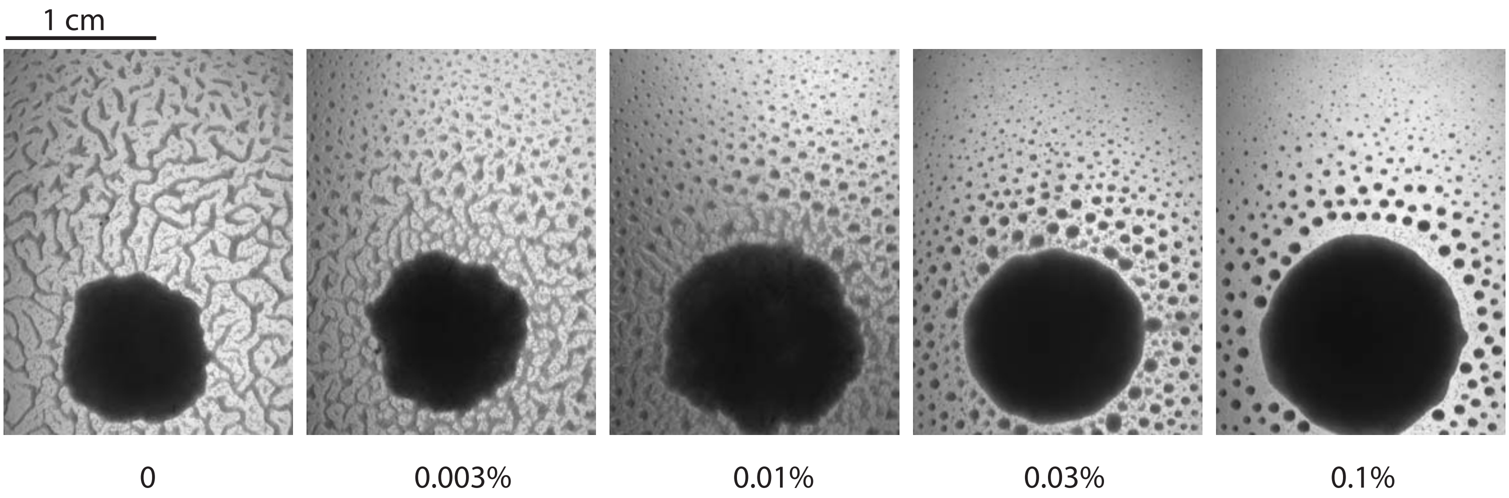

Fig. S4. Aggregation of starved N2 L1s at various ethanol concentrations in agarose plates. Images were taken 23 h after beginning of the aggregation experiment.

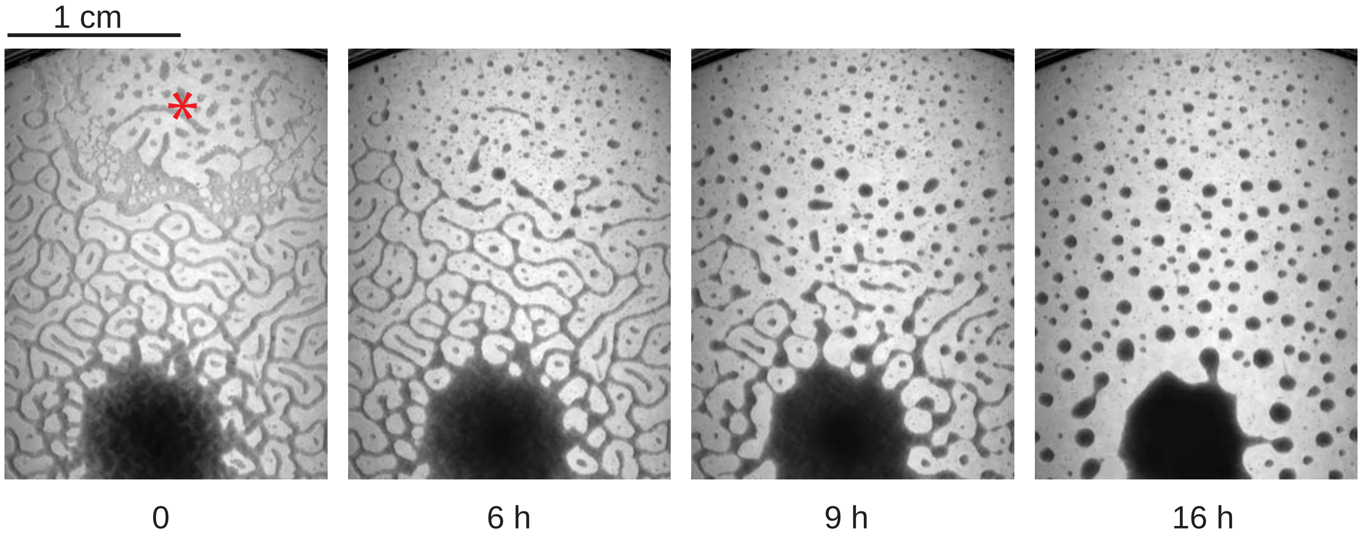

Fig. S5. Addition of ethanol induces L1 aggregation even on the plate, where L1s were pipetted 9 days ago. 5 μl of ethanol was added to a side of a 6cm plate with L1s (red asterisk).

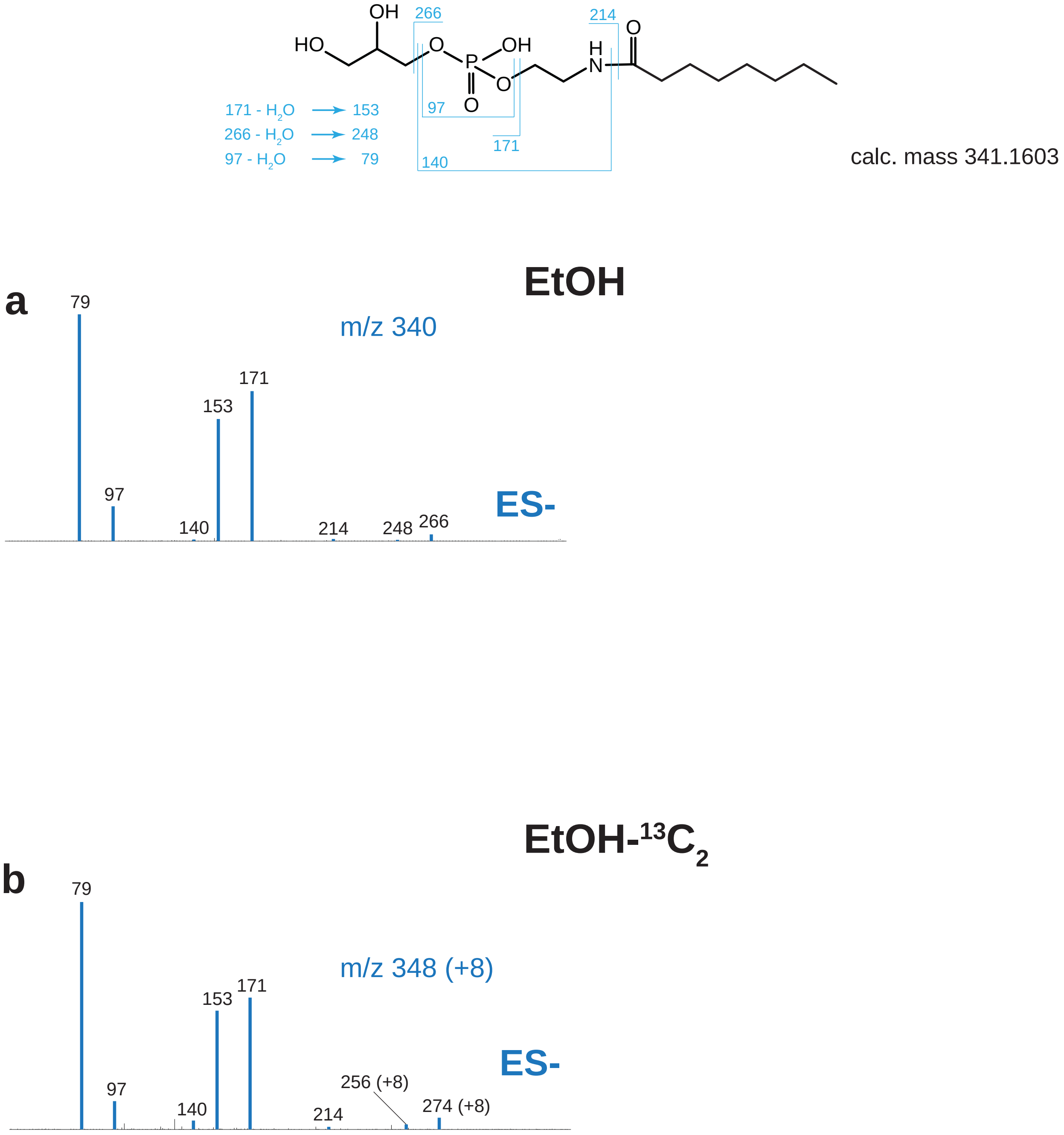

Fig. S6. Mass spectra of natural **ea#8** from L1 medium conditioned with regular ethanol (a) or ^{13}C-labeled ethanol (b). Major fragmentation reactions corresponding to observed ions are indicated in the structure.

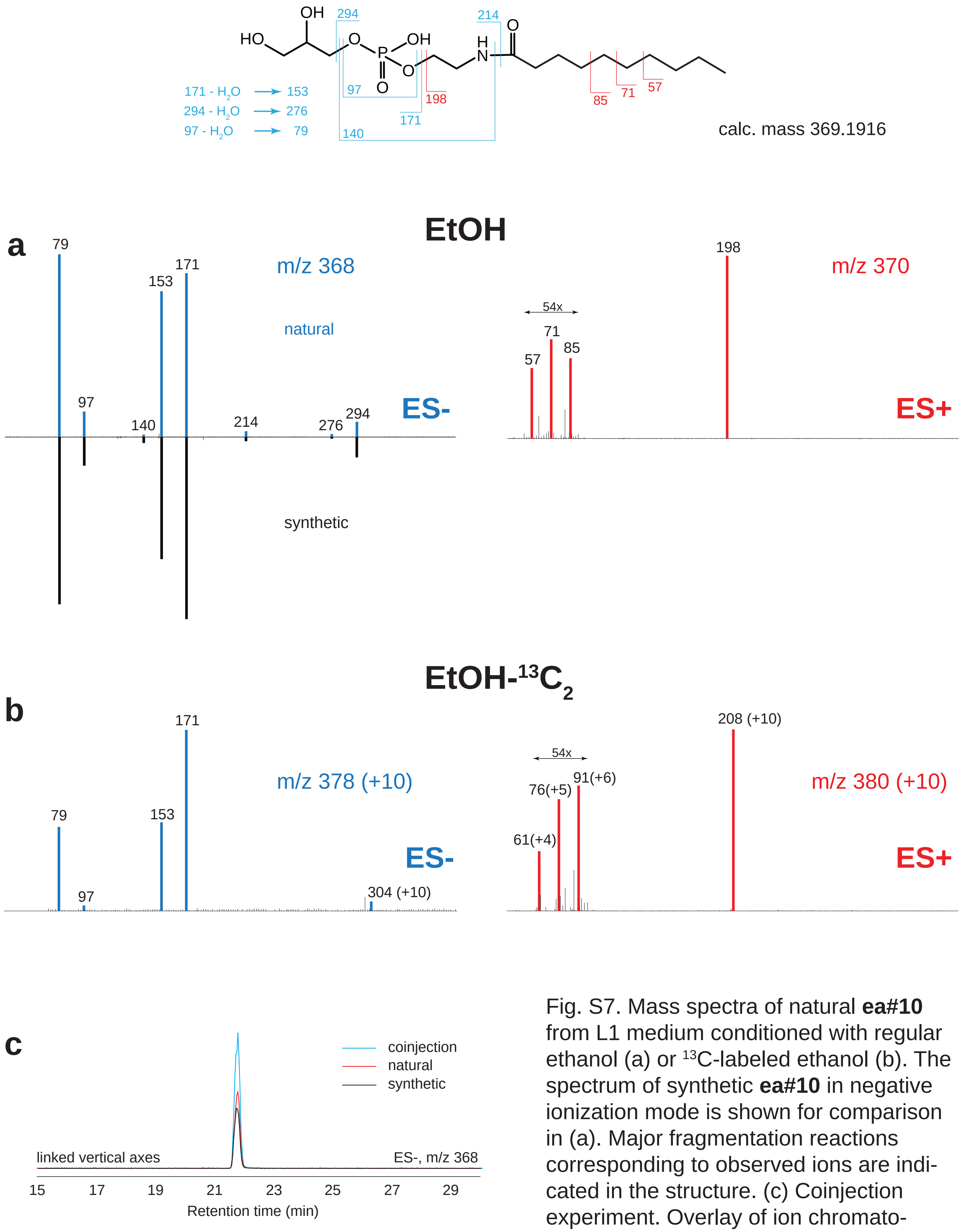

Fig. S7. Mass spectra of natural **ea#10** from L1 medium conditioned with regular ethanol (a) or ^{13}C-labeled ethanol (b). The spectrum of synthetic **ea#10** in negative ionization mode is shown for comparison in (a). Major fragmentation reactions corresponding to observed ions are indicated in the structure. (c) Coinjection experiment. Overlay of ion chromatograms of natural **ea#10** from L1 conditioned medium and synthetic **ea#10** along with 1:1 mixture of the two.

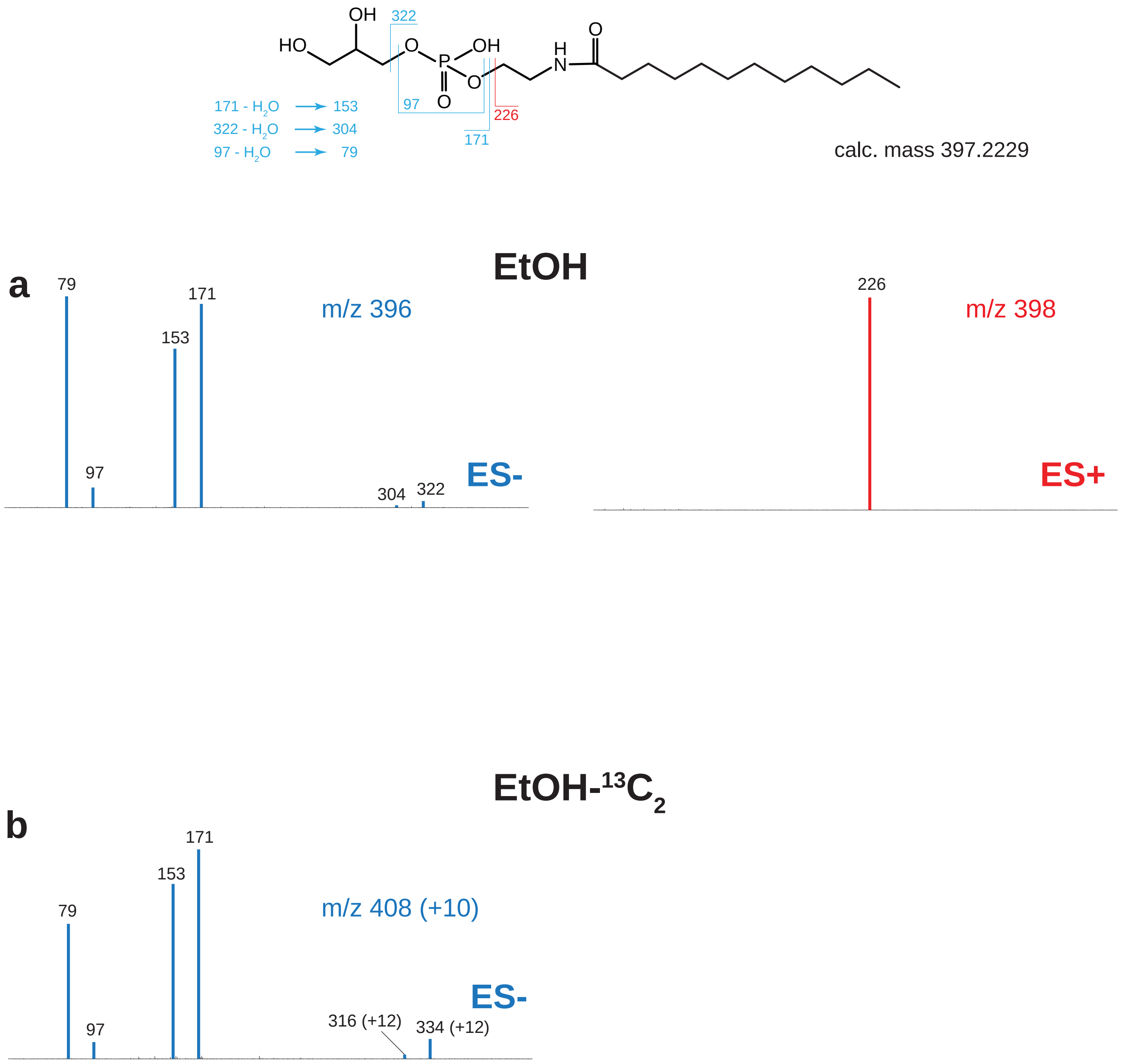

Fig. S8. Mass spectra of natural **ea#12** from L1 medium conditioned with regular ethanol (a) or ^{13}C-labeled ethanol (b). Major fragmentation reactions corresponding to observed ions are indicated in the structure.

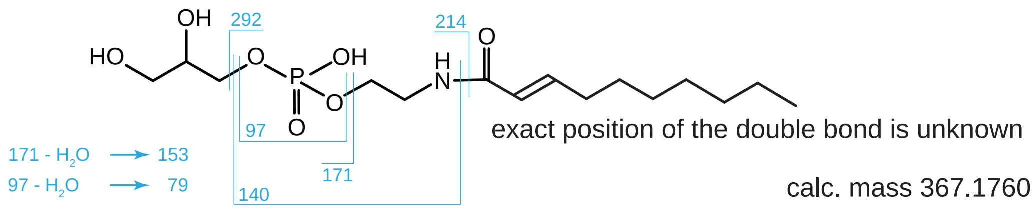

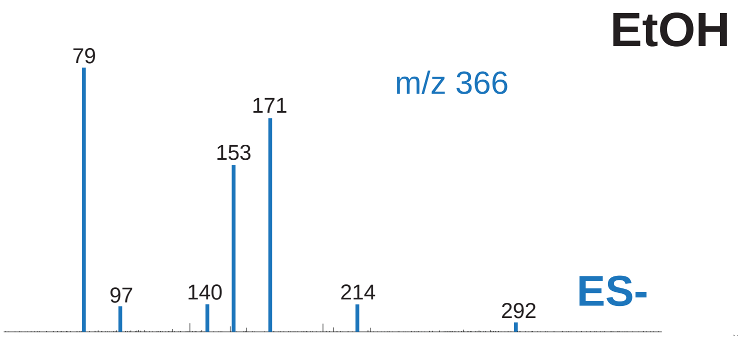

Fig. S9. Mass spectra of natural Δ**ea#10** from L1 medium conditioned with regular ethanol. Major fragmentation reactions corresponding to observed ions are indicated in the structure.

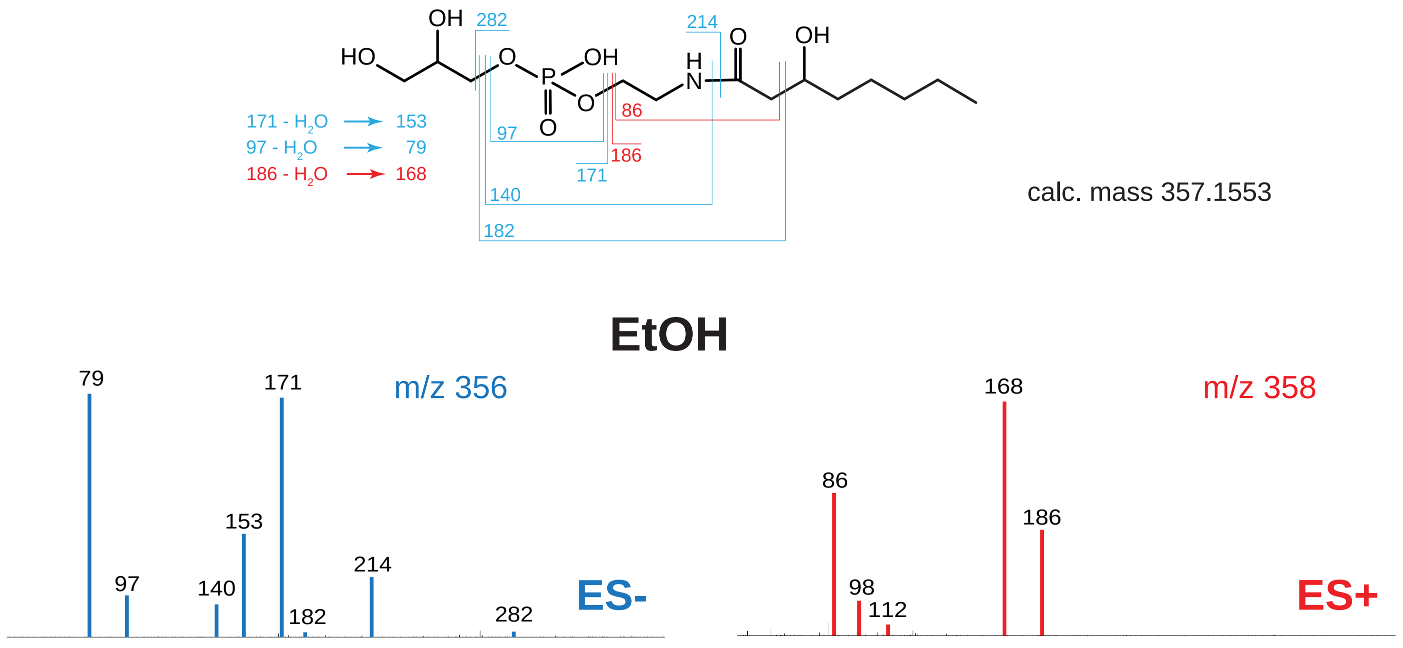

Fig. S10. Mass spectra of natural **hea#8** from L1 medium conditioned with regular ethanol. Major fragmentation reactions corresponding to observed ions are indicated in the structure.

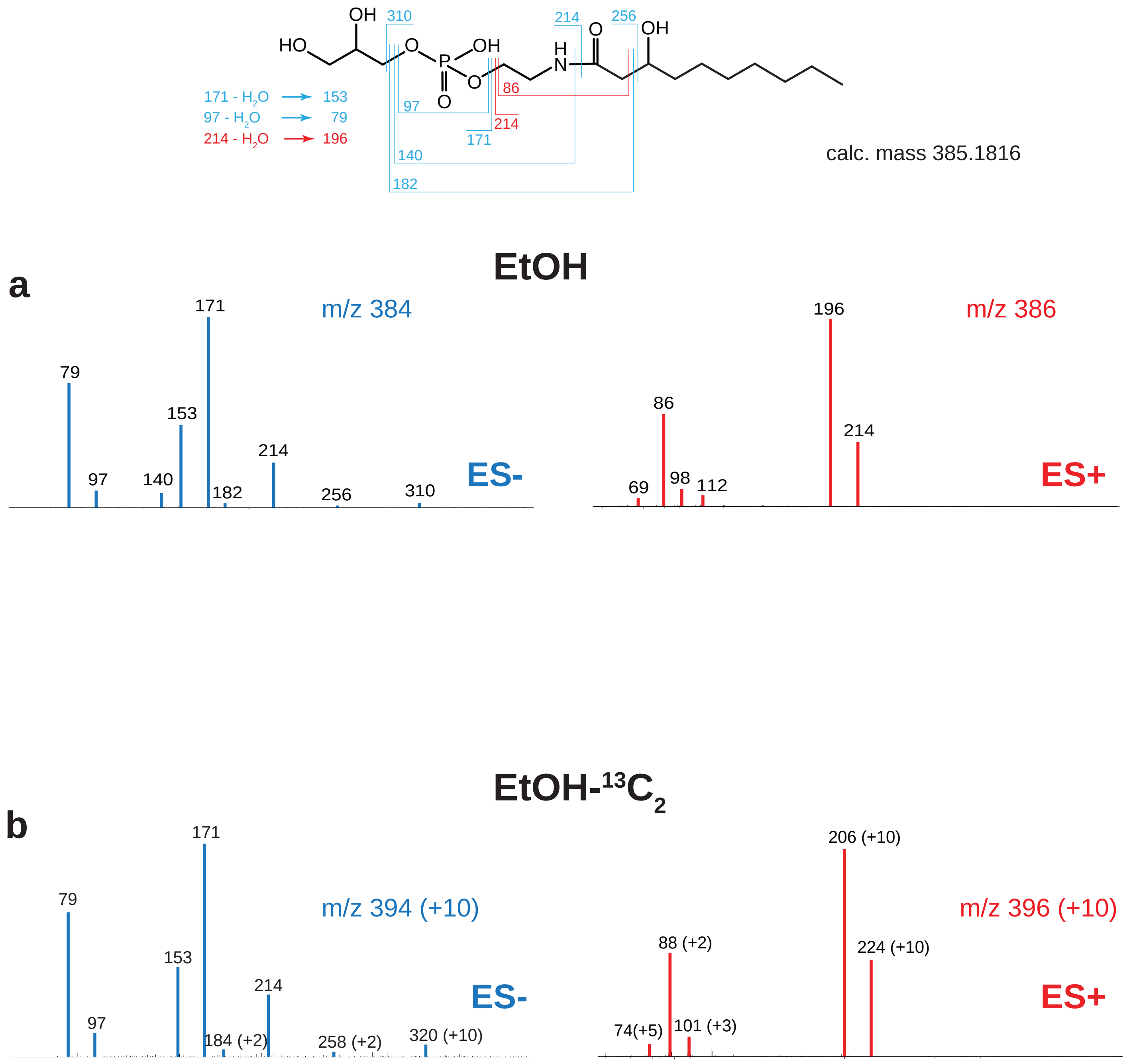

Fig. S11. Mass spectra of natural **hea#10** from L1 medium conditioned with regular ethanol (a) or ^{13}C-labeled ethanol (b). Major fragmentation reactions corresponding to observed ions are indicated in the structure.

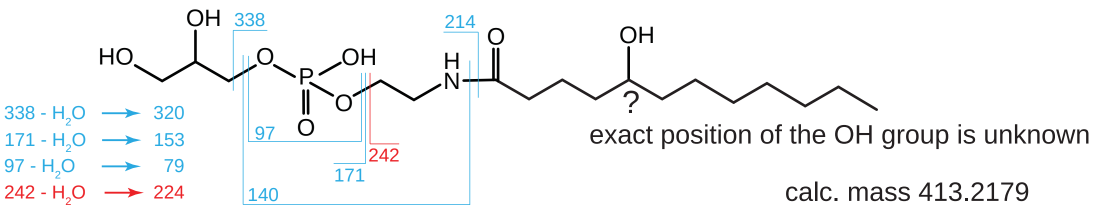

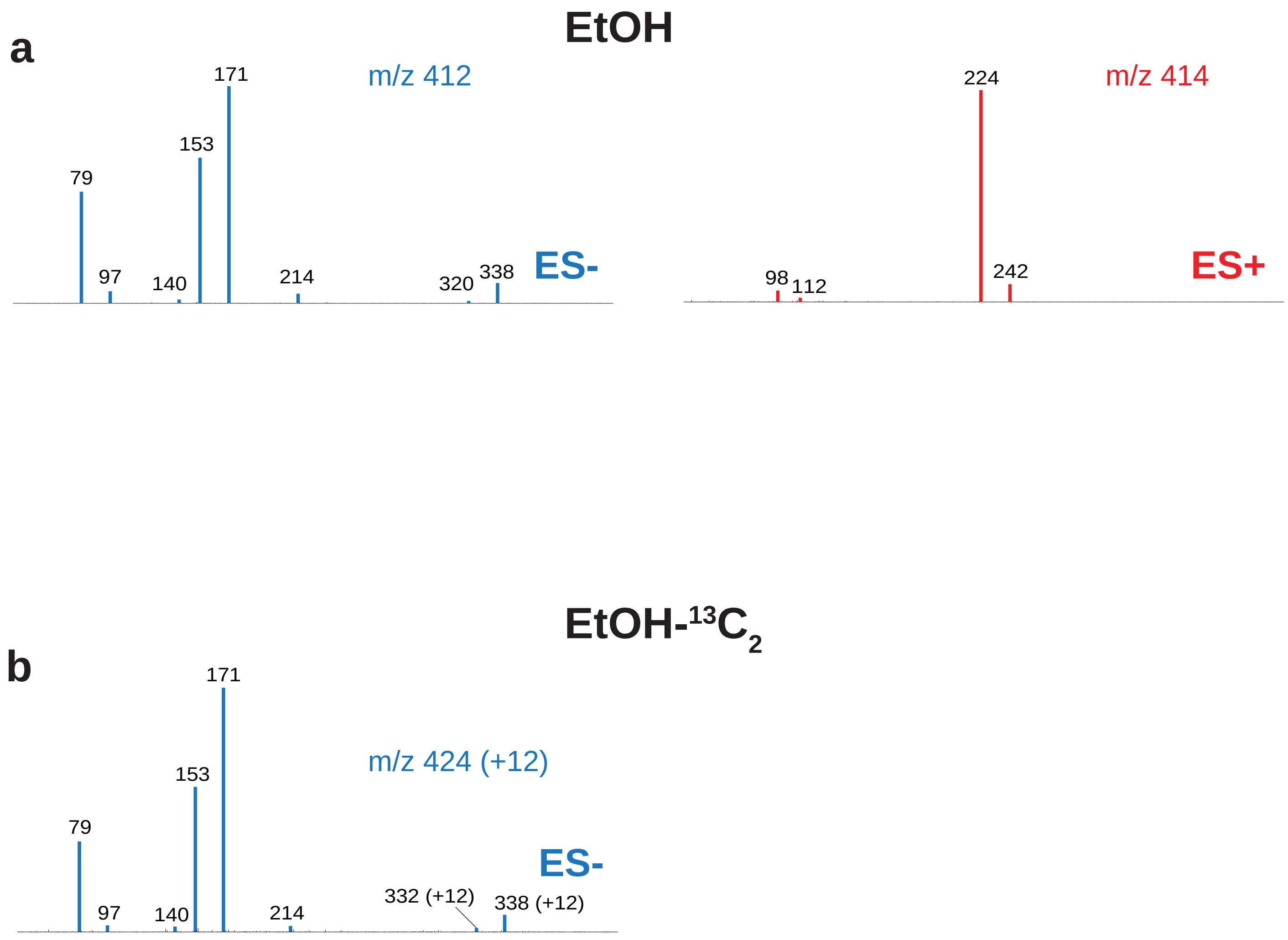

Fig. S12. Mass spectra of natural **hea#12.1** from L1 medium conditioned with regular ethanol (a) or ^{13}C-labeled ethanol (b). Major fragmentation reactions corresponding to observed ions are indicated in the structure.

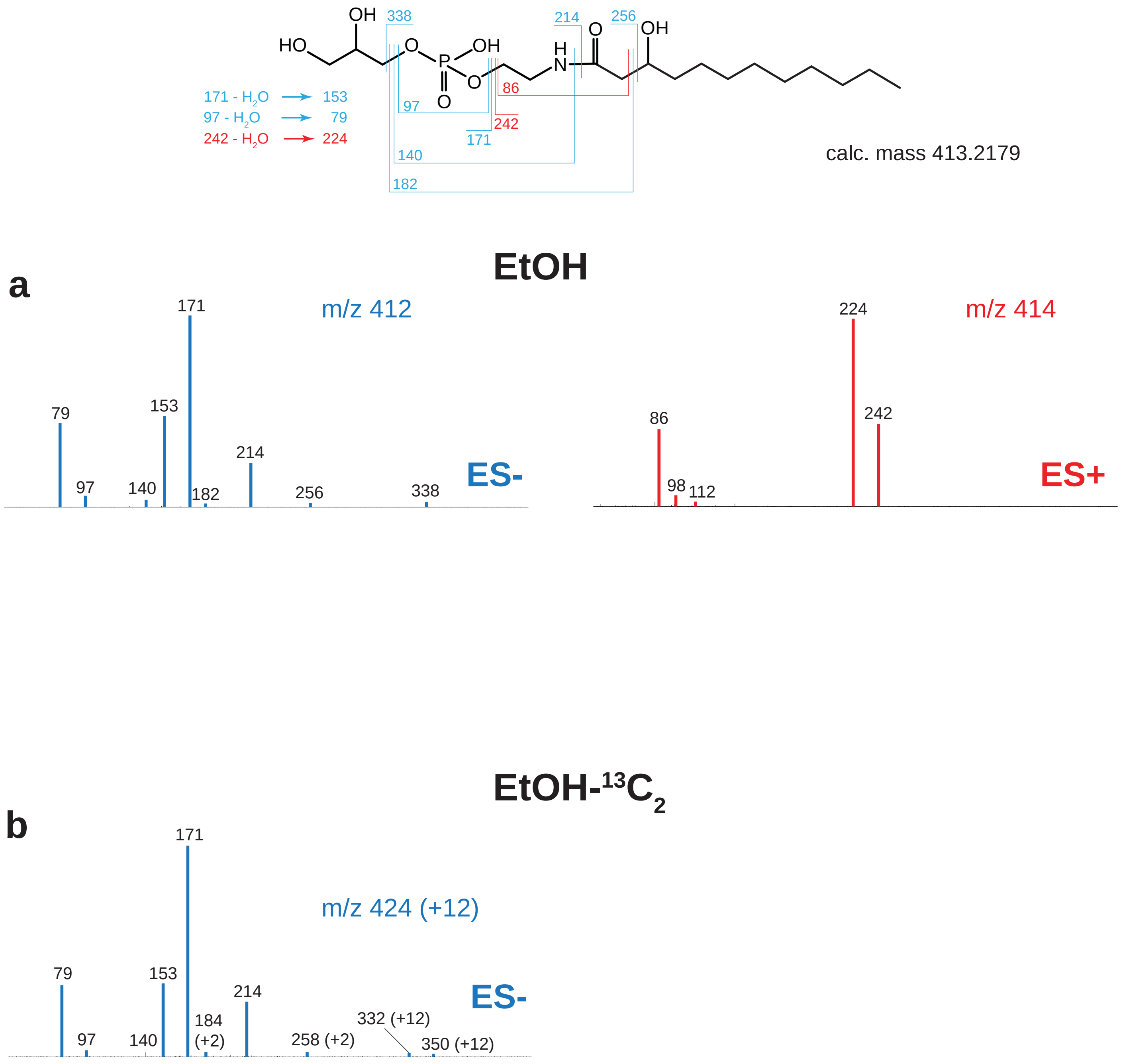

Fig. S13. Mass spectra of natural **hea#12.2** from L1 medium conditioned with regular ethanol (a) or ^{13}C-labeled ethanol (b). Major fragmentation reactions corresponding to observed ions are indicated in the structure.

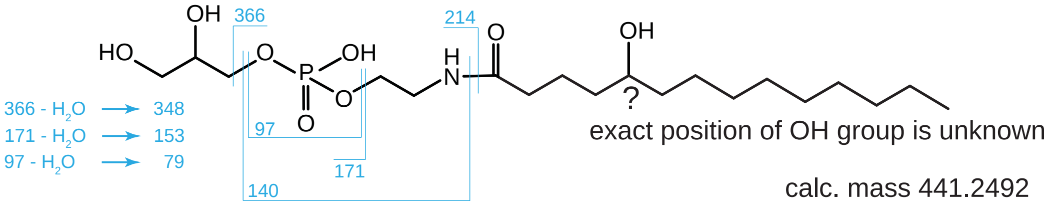

EtOH

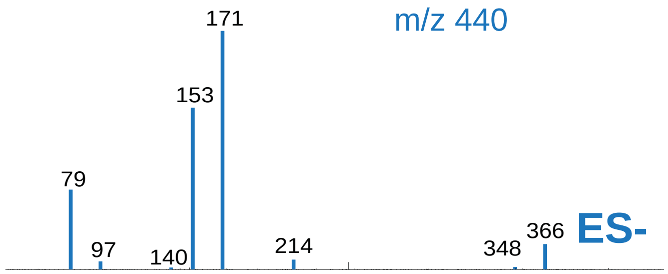

Fig. S14. Mass spectra of natural **hea#14** from L1 medium conditioned with regular ethanol. Major fragmentation reactions corresponding to observed ions are indicated in the structure.

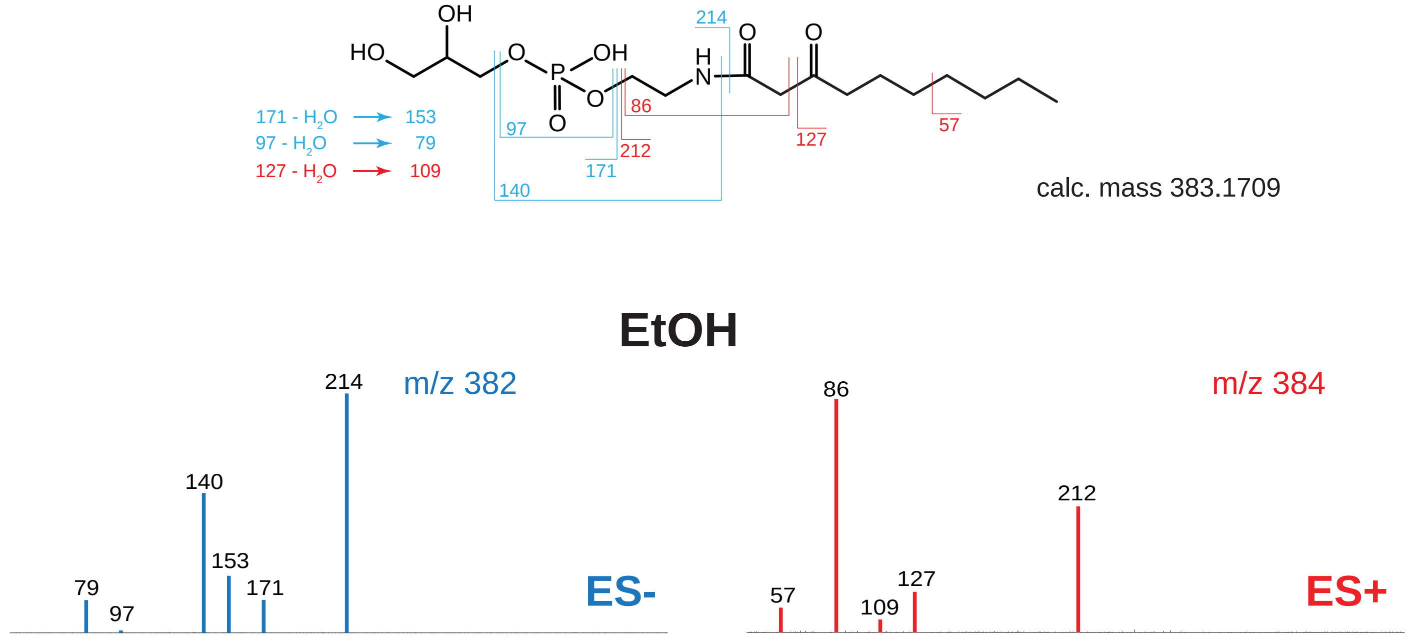

Fig. S15. Mass spectra of natural **oxea#10** from L1 medium conditioned with regular ethanol. Major fragmentation reactions corresponding to observed ions are indicated in the structure.

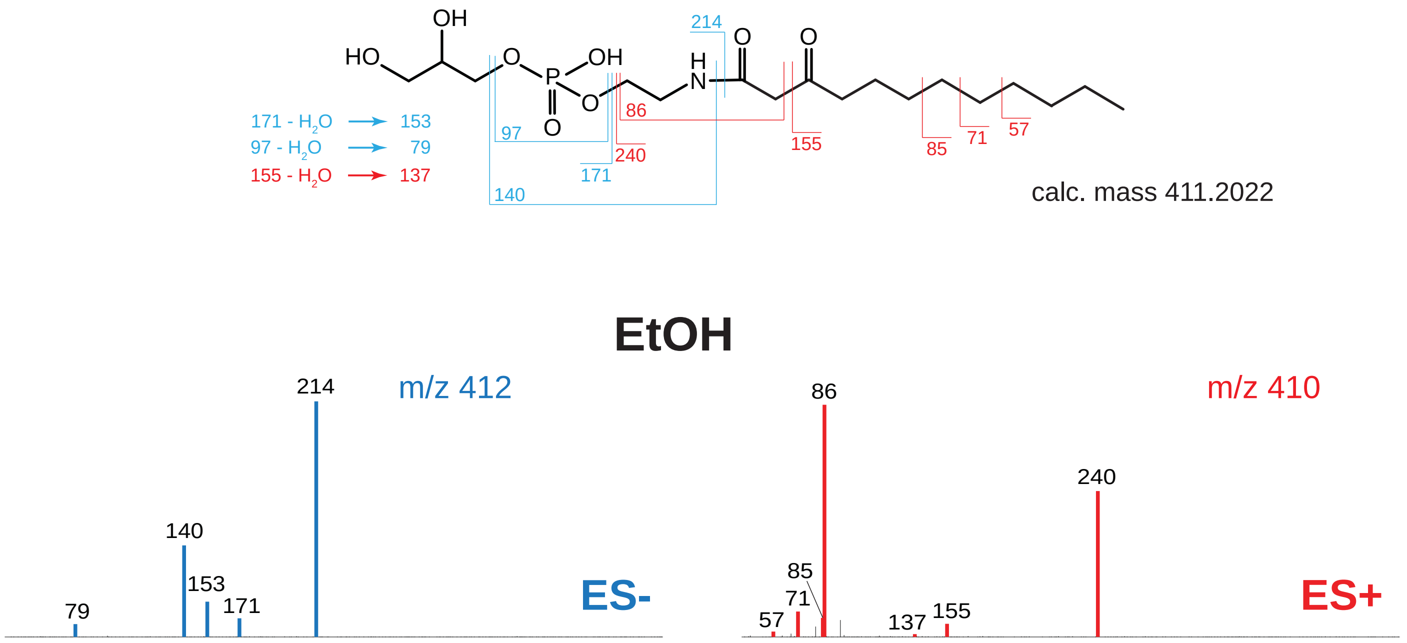

Fig. S16. Mass spectra of natural **oxea#12** from L1 medium conditioned with regular ethanol. Major fragmentation reactions corresponding to observed ions are indicated in the structure.

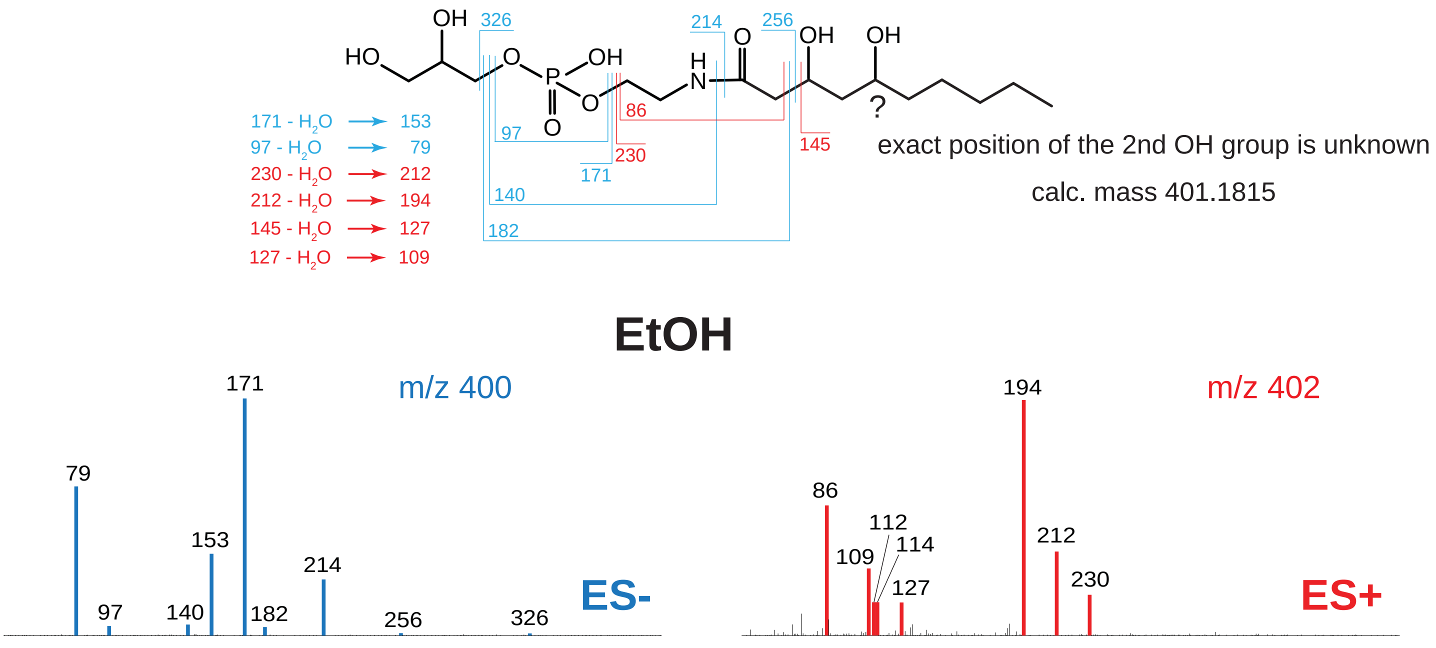

Fig. S17. Mass spectra of natural **dhea#10** from L1 medium conditioned with regular ethanol. Major fragmentation reactions corresponding to observed ions are indicated in the structure.

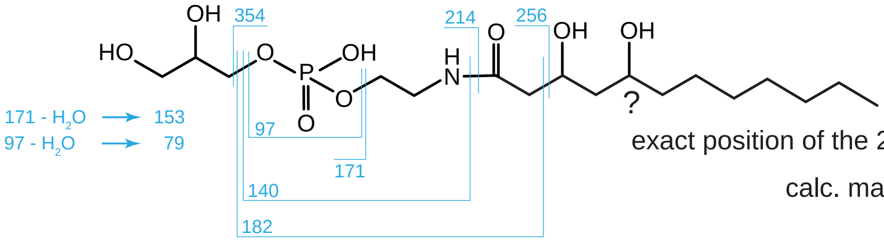

exact position of the 2nd OH group is unknown

calc. mass 429.2128

EtOH

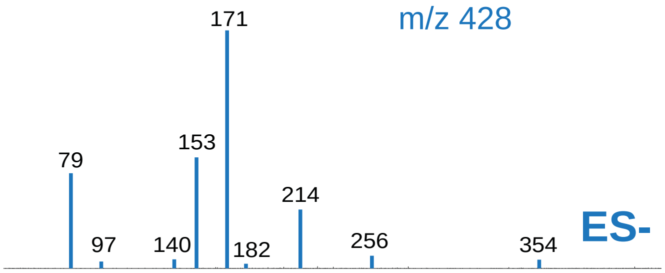

Fig. S18. Mass spectra of natural **dhea#12** from L1 medium conditioned with regular ethanol. Major fragmentation reactions corresponding to observed ions are indicated in the structure.

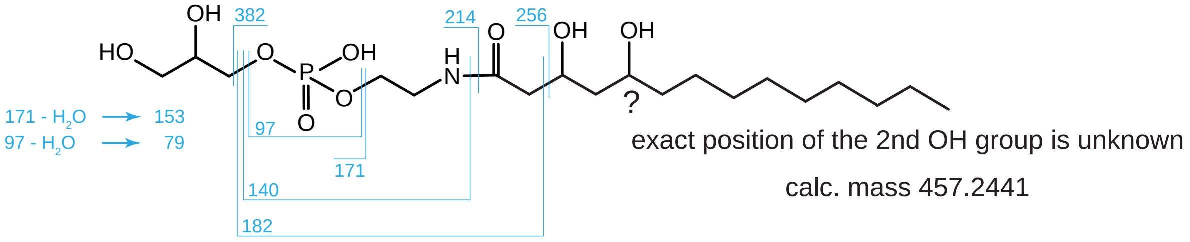

EtOH

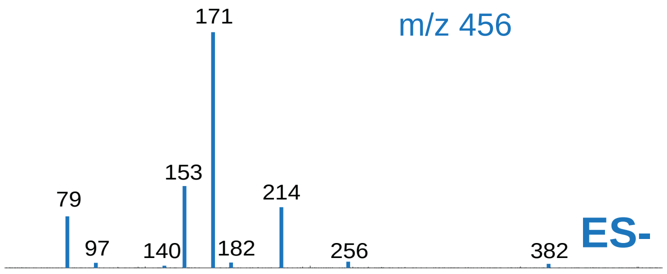

Fig. S19. Mass spectra of natural **dhea#14** from L1 medium conditioned with regular ethanol. Major fragmentation reactions corresponding to observed ions are indicated in the structure.

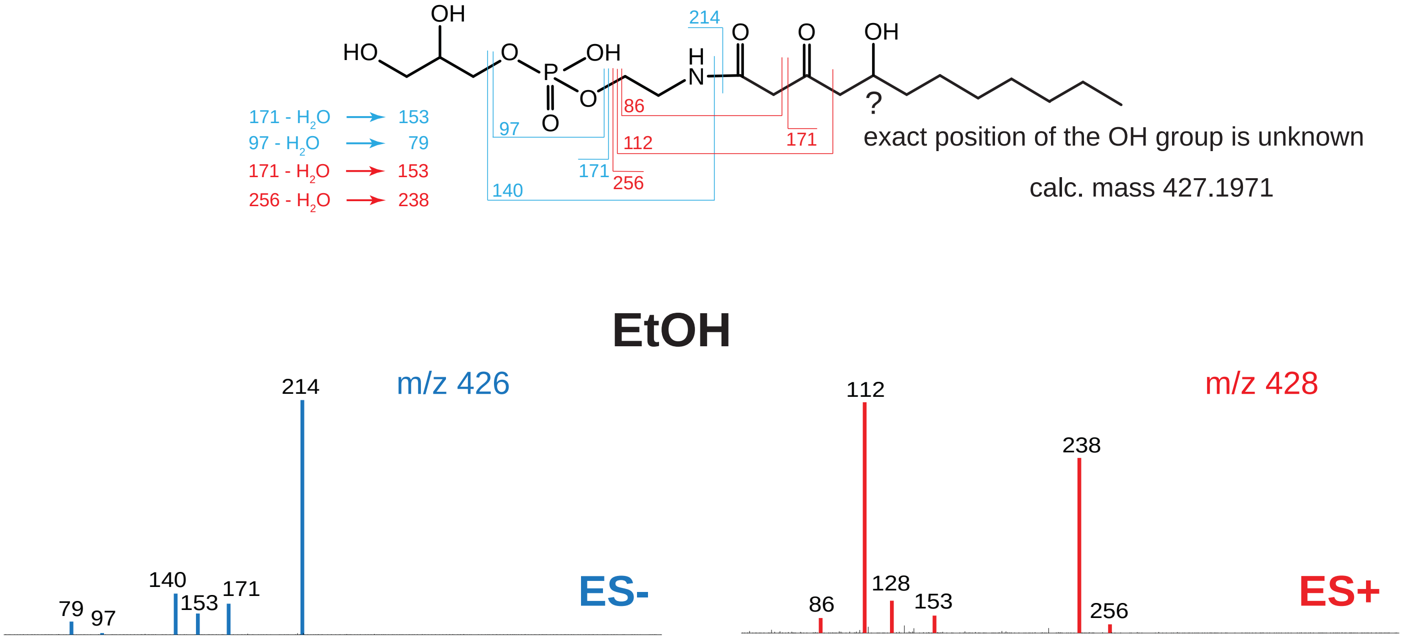

Fig. S20. Mass spectra of natural **ohea#12** from L1 medium conditioned with regular ethanol. Major fragmentation reactions corresponding to observed ions are indicated in the structure.

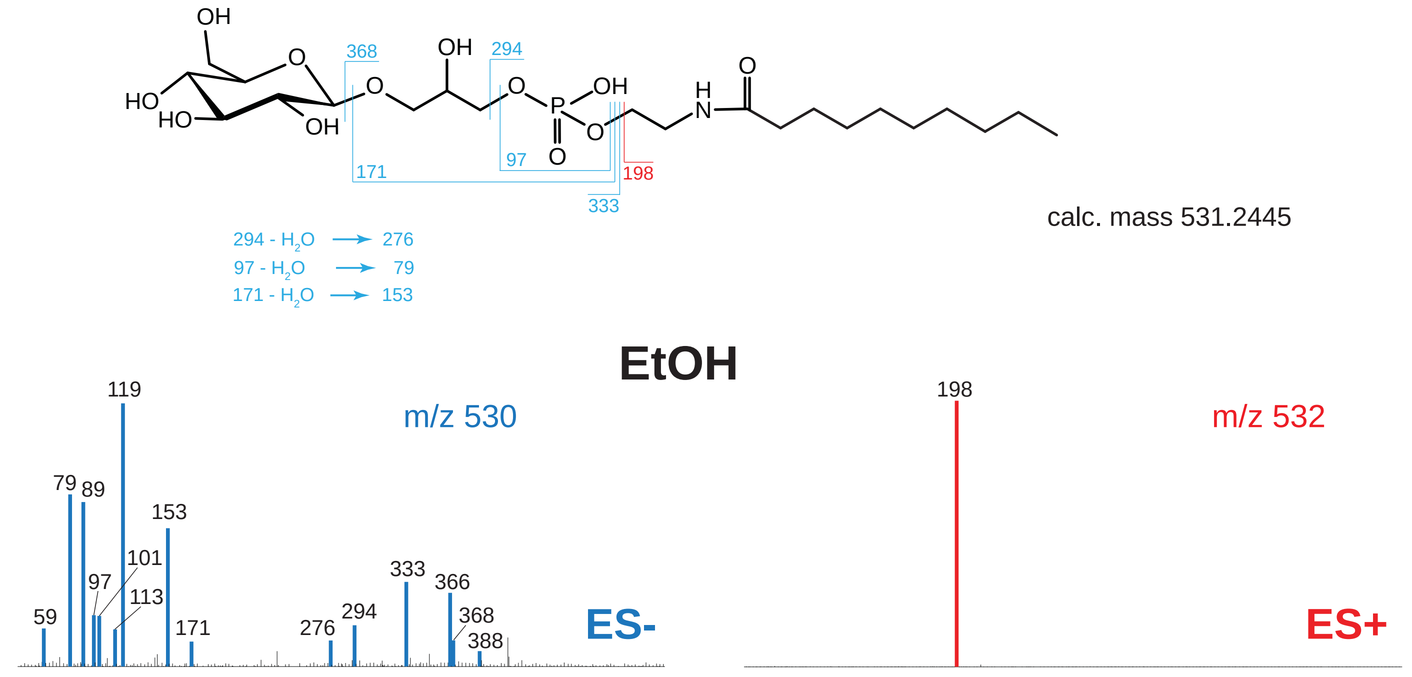

m/z 59, 89, 101, 113, 119 are glucose fragments

Fig. S21. Mass spectra of natural **glea#10** from L1 medium conditioned with regular ethanol. Major fragmentation reactions corresponding to observed ions are indicated in the structure. The glucose moiety is assumed to be β-D-glucose attached through 1-hydroxy group, as is often the case in *C. elegans* glucosides, but the exact stereochemistry has not been verified.

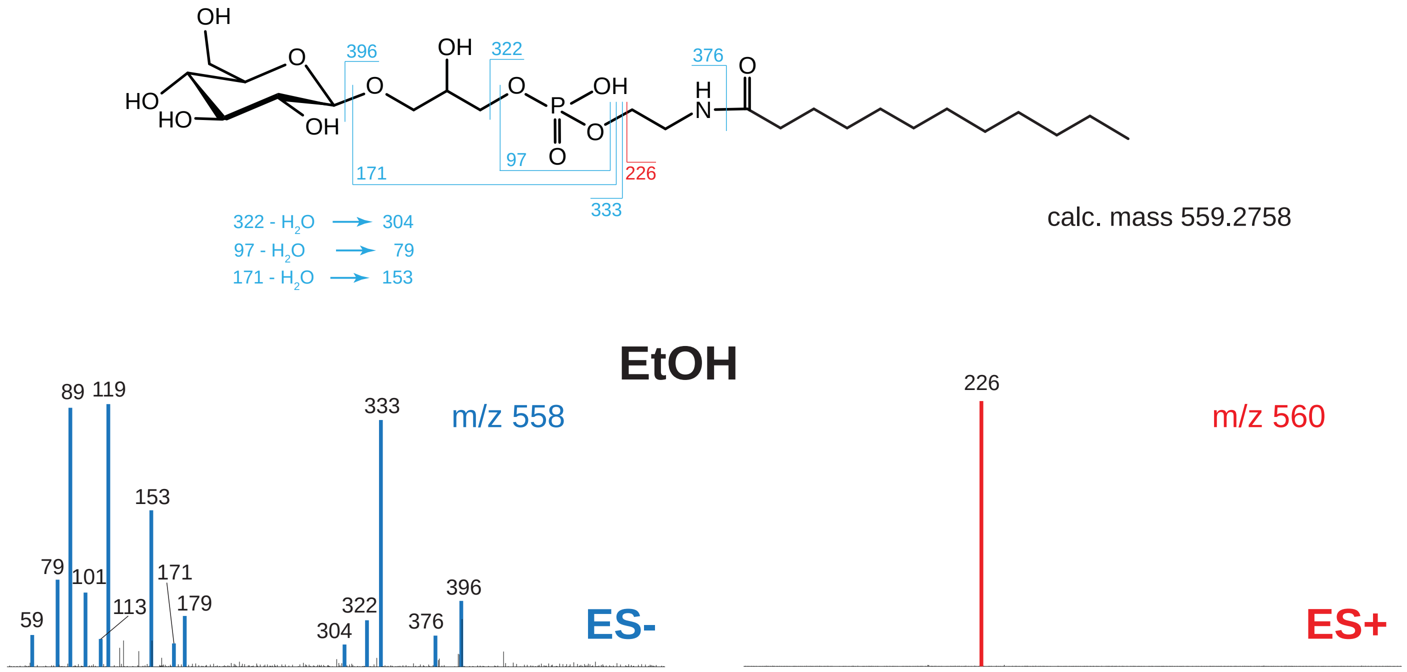

m/z 59, 89, 101, 113, 119, 179 are glucose fragments

Fig. S22. Mass spectra of natural **glea#12** from L1 medium conditioned with regular ethanol. Major fragmentation reactions corresponding to observed ions are indicated in the structure. The glucose moiety is assumed to be β-D-glucose attached through 1-hydroxy group, as is often the case in *C. elegans* glucosides, but the exact stereochemistry has not been verified.

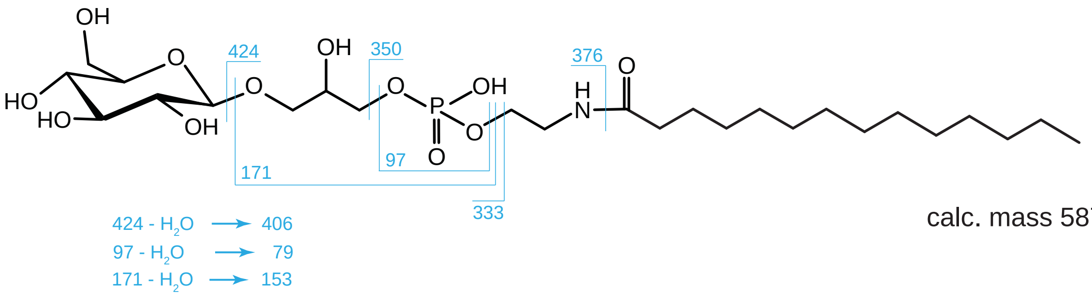

calc. mass 587.3071

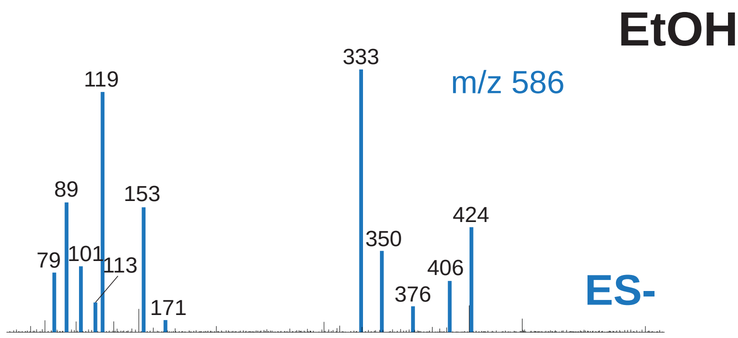

m/z 89, 101, 113, 119 are glucose fragments

Fig. S23. Mass spectra of natural **glea#14** from L1 medium conditioned with regular ethanol. Major fragmentation reactions corresponding to observed ions are indicated in the structure. The glucose moiety is assumed to be β-D-glucose attached through 1-hydroxy group, as is often the case in *C. elegans* glucosides, but the exact stereochemistry has not been verified.

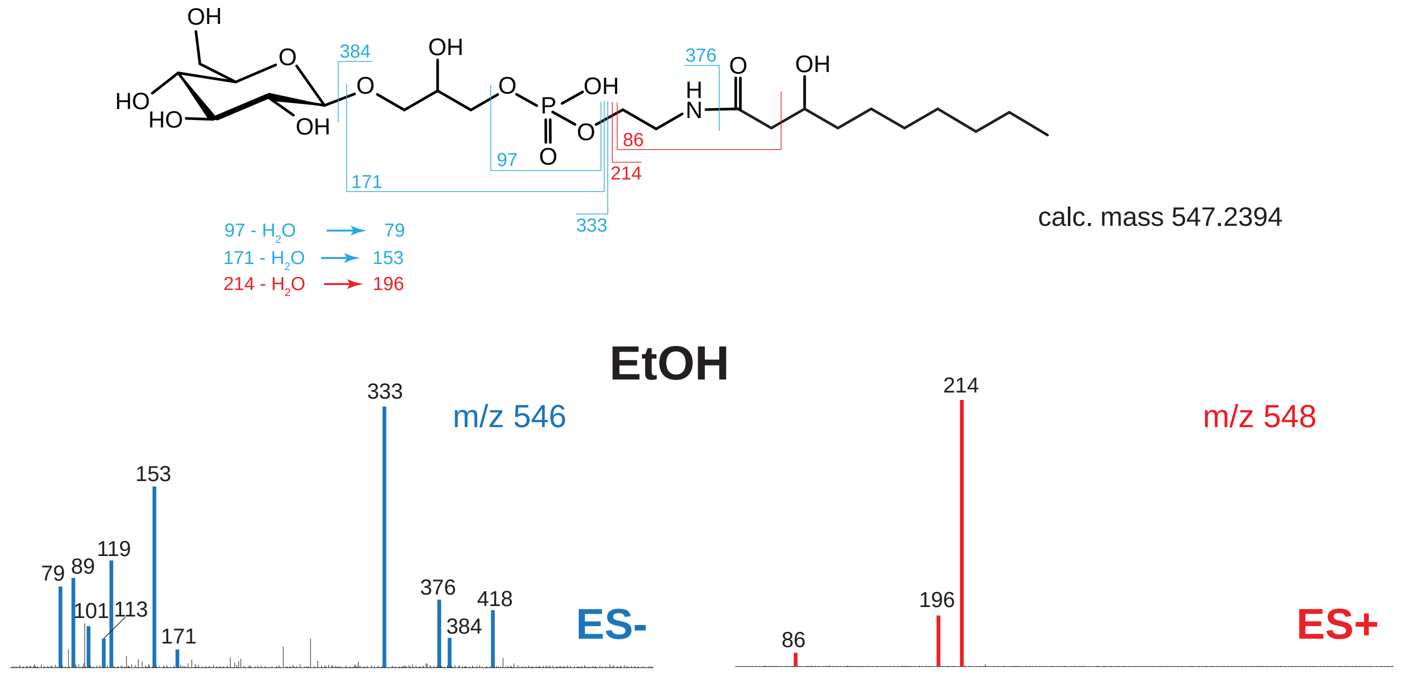

m/z 89, 101, 113, 119 are glucose fragments

Fig. S24. Mass spectra of natural **ghea#10** from L1 medium conditioned with regular ethanol. Major fragmentation reactions corresponding to observed ions are indicated in the structure. The glucose moiety is assumed to be β-D-glucose attached through 1-hydroxy group, as is often the case in *C. elegans* glucosides, but the exact stereochemistry has not been verified.

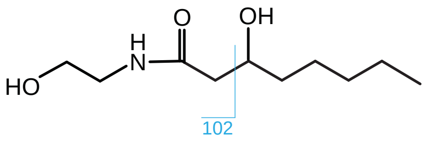

calc. mass 203.1521

EtOH

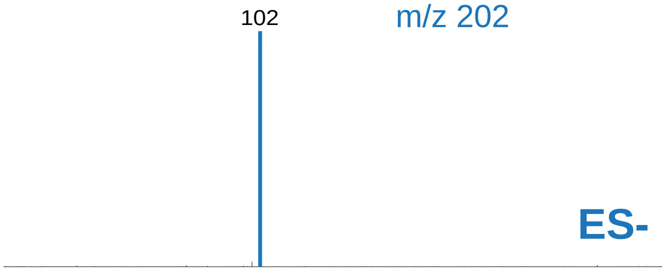

Fig. S25. Mass spectra of natural **shea#10** from L1 medium conditioned with regular ethanol. Major fragmentation reaction corresponding to the observed ion is indicated in the structure.
